# Human fertilization in vivo and in vitro requires the CatSper channel to initiate sperm hyperactivation

**DOI:** 10.1172/JCI173564

**Published:** 2024-01-02

**Authors:** Samuel Young, Christian Schiffer, Alice Wagner, Jannika Patz, Anton Potapenko, Leonie Herrmann, Verena Nordhoff, Tim Pock, Claudia Krallmann, Birgit Stallmeyer, Albrecht Röpke, Michelina Kierzek, Cristina Biagioni, Tao Wang, Lars Haalck, Dirk Deuster, Jan N. Hansen, Dagmar Wachten, Benjamin Risse, Hermann M. Behre, Stefan Schlatt, Sabine Kliesch, Frank Tüttelmann, Christoph Brenker, Timo Strünker

**Affiliations:** 1 Centre of Reproductive Medicine and Andrology, University Hospital Münster, University of Münster, Münster, Germany.; 2 Institute of Reproductive Genetics,; 3 Institute of Human Genetics,; 4 CiM-IMPRS Graduate School,; 5 Institute of Geoinformatics, Computer Vision and Machine Learning Systems, University of Münster, Münster, Germany.; 6 Department of Phoniatrics and Pedaudiology, University Hospital Münster, University of Münster, Münster, Germany.; 7 Institute of Innate Immunity, Department of Biophysical Imaging, Medical Faculty, University of Bonn, Bonn, Germany.; 8 Computer Science Department, University of Münster, Münster, Germany.; 9 UKM Fertility Centre, University Hospital Münster, Münster, Germany.; 10 Cells in Motion Interfaculty Centre, University of Münster, Münster, Germany.

**Keywords:** Cell Biology, Reproductive Biology, Calcium channels, Fertility

## Abstract

The infertility of many couples rests on an enigmatic dysfunction of the man’s sperm. To gain insight into the underlying pathomechanisms, we assessed the function of the sperm-specific multisubunit CatSper-channel complex in the sperm of almost 2,300 men undergoing a fertility workup, using a simple motility-based test. We identified a group of men with normal semen parameters but defective CatSper function. These men or couples failed to conceive naturally and upon medically assisted reproduction via intrauterine insemination and in vitro fertilization. Intracytoplasmic sperm injection (ICSI) was, ultimately, required to conceive a child. We revealed that the defective CatSper function was caused by variations in *CATSPER* genes. Moreover, we unveiled that CatSper-deficient human sperm were unable to undergo hyperactive motility and, therefore, failed to penetrate the egg coat. Thus, our study provides the experimental evidence that sperm hyperactivation is required for human fertilization, explaining the infertility of CatSper-deficient men and the need of ICSI for medically assisted reproduction. Finally, our study also revealed that defective CatSper function and ensuing failure to hyperactivate represents the most common cause of unexplained male infertility known thus far and that this sperm channelopathy can readily be diagnosed, enabling future evidence-based treatment of affected couples.

## Abstract 2

Defective CatSper function represents a common channelopathy affecting male fertility

## Graphical abstract

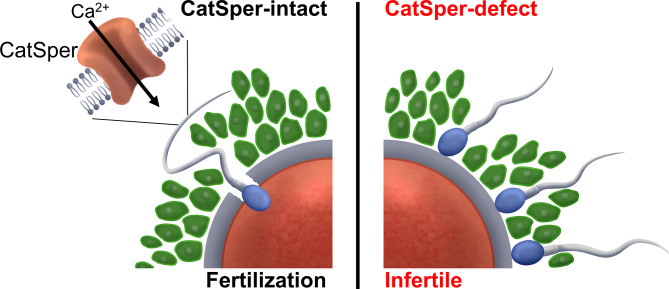

## Introduction

Infertility is on the rise, but the underlying causes still remain mostly unknown. In fact, for about a third of infertile men, semen parameters are within reference limits (normozoospermia) (1). This so called unexplained male infertility seems to rest on elusive functional defects of the sperm undetectable by semen analysis, precluding early diagnosis of the infertility and evidence-based treatment by medically assisted reproduction (MAR) (2). As a result, affected couples often experience failed MAR attempts, subjecting the healthy woman to recurring medical risks. To address this issue, we must elucidate the pathomechanisms underlying unexplained male infertility and, thereby, identify the molecules that orchestrate human sperm function and fertilization.

Most sperm functions, e.g., the swimming behavior (3, 4), are controlled by changes in the intracellular Ca^2+^ concentration ([Ca^2+^]_i_). In sperm from many species including mammals, [Ca^2+^]_i_ is controlled by the sperm-specific Ca^2+^ channel CatSper (5–13). Mammalian CatSper comprises 4 pore-forming subunits (CatSper 1–4) and a large number of auxiliary subunits (CatSper β, -γ, -δ, -ɛ, -ζ, -η, -τ, Trim69, Slco6c1, Efcab9, Tmem249 and, perhaps, Cdc42) (14–21), rendering CatSper the most complex ion channel known (see Supplemental Table 1 for official NCBI protein/gene nomenclature in mice and humans; supplemental material available online with this article; https://doi.org/10.1172/JCI173564DS1). In mice, targeted ablation of genes encoding CatSper subunits does not affect sperm number, morphology, or basal motility (5, 16–18, 22–25). However, mouse sperm lacking CatSper are unable to switch to so-called hyperactivated motility (16, 22, 24, 26–29), i.e., a powerful flagellar beat characterized by high asymmetry and low frequency required for mouse sperm to penetrate the egg coat (5, 27, 28, 30). Therefore, loss of CatSper function results in male infertility in mice (9). This suggests that unexplained male infertility in humans might involve defective CatSper function and/or failure of sperm to hyperactivate.

Genetic analysis of infertile men primarily from consanguineous families with clustering of male-factor infertility, indeed, identified individuals with nonsense variants in the *CATSPER1* or *CATSPER3* genes as well as with a homozygous deletion of *CATSPER2* and the contiguous gene *STRC* (31–36). The latter was termed deafness-infertility syndrome, or DIS (OMIM no. 611102) (33), because deletion of *STRC* causes mild-to-moderate hearing loss (37–39). Two additional men with variants in *CATSPERE* (40, 41) or *CATSPER2* (42) were identified by functional analysis of CatSper in sperm from infertile men.

Remarkably, only a few men with variants in *CATSPER* genes presumed or experimentally proven to cause loss of CatSper function have thus far been examined in detail, and the individual studies indicated different phenotypes; 3 men were reported as normozoospermic (36, 40, 42), but their sperm seemingly featured different functional defects. In particular, hyperactivated motility was either abolished (42), unaffected (40), or not even assessed (36). Another 7 men were reported as (oligo)asthenoteratozoospermic (31–35), i.e., their ejaculate contained only few, poorly motile and morphological abnormal sperm, explaining their infertility and suggesting that in humans, as opposed to mice, CatSper is required for spermatogenesis.

Thus, since its discovery in mice more than 2 decades ago, the role of CatSper in spermatogenesis and sperm hyperactivation in humans as well as the role of sperm hyperactivation in human fertilization and male infertility remain to be elucidated (10).

To this end, we developed a ‘CatSper-Activity-Test’, which we used in combination with [Ca^2+^]_i_ fluorimetric and electrophysiological recordings to systematically assess the function of CatSper in sperm of thousands of men undergoing a fertility workup at our center. Thereby, we identified 8 men with loss of CatSper function featuring homozygous deletions of *CATSPER2* and a man with impaired channel function featuring biallelic variants of *CATSPERE*. We investigated the phenotype of these men and their sperm, using novel microfluidic and optochemical techniques for motility and flagellar-beat analyses as well as video-microscopic monitoring of MAR via in vitro fertilization (IVF) and intracytoplasmic sperm injection (ICSI). Together, this unveiled that CatSper is not involved in human spermatogenesis but is required to initiate hyperactivation of human sperm. Moreover, our results indicate that hyperactivation initiated by CatSper is required for human sperm to penetrate the egg coat and, thus, for human fertilization in vivo and in vitro. Finally, we revealed that defective CatSper function and ensuing failure to hyperactivate represents the most common cause of unexplained male-factor infertility know thus far with an estimated prevalence of 2.3% for this sperm channelopathy in this group of patients.

## Results

### The activity of CatSper in human sperm can be determined by a simple motility-based test.

**Figure 1:**
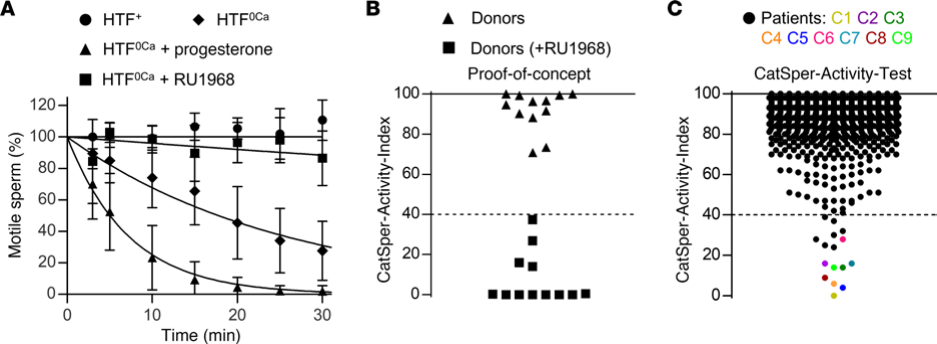
Development of a motility-based test to assess the activity of CatSper in human sperm. (**A**) Changes in the fraction of motile sperm (mean ± SD) upon dilution of semen samples from donors in Ca^2+^-free HTF (HTF^0Ca^) (diamonds; *n* = 9), HTF^0Ca^ containing progesterone (10 μM) (triangles; *n* = 9), or HTF^0Ca^ containing the CatSper-inhibitor RU1968 (15 μM) (squares; *n* = 6), relative to the fraction of motile sperm determined upon dilution of the respective semen sample in control HTF^+^ (circles, *n* = 9) at *t* = 0 (set to 100%). An exponential decay curve was fitted to the change in the fraction of motile cells averaged over all replicates. (**B**) CatSper-Activity-Indices (CAI) determined 15 minutes after dilution of semen samples from donors (*n* = 12) in HTF^+^ (Buffer A) and HTF^0Ca^ containing progesterone (triangles) (Buffer B) or Buffer B also containing RU1968 (15 μM) (squares). (**C**) CAI values from semen samples of men undergoing semen analysis (*n* = 2,286); the dotted line indicates the CAI threshold, i.e., values above and below were considered indicative of normal and defective CatSper function, respectively. Patients with confirmed loss or impaired CatSper function (see Figure 2) are labeled C1–C9 and indicated with a color-coded circle.

To elucidate the role of CatSper in human fertilization, we set out to develop a laboratory test that identifies patients with defective CatSper function. Lowering the extracellular Ca^2+^ concentration ([Ca^2+^]_o_) to nanomolar levels renders mouse (27) and human sperm (43, 44) immotile. In mouse sperm lacking CatSper, the cessation of motility by low [Ca^2+^]_o_ is abolished (27), demonstrating that it requires functional CatSper. We tested whether this holds true for human sperm. An aliquot of the ejaculate from donors with normal CatSper function was diluted tenfold with control (HTF^+^) or Ca^2+^-free HTF buffer (HTF^0Ca^, [Ca^2+^]_o_ < 20 nM), and the ensuing change in the fraction of motile sperm was monitored over time. In control buffer, the fraction of motile sperm remained constant, whereas in Ca^2+^-free buffer, it decreased exponentially with a time constant (τ) of 27 ± 11 minutes (mean ± SD, *n* = 9) (Figure 1A). In Ca^2+^-free buffer containing the CatSper-inhibitor RU1968 (45), the decrease was abolished (Figure 1A), indicating that it requires functional CatSper. Of note, in human sperm, CatSper is activated by steroids and prostaglandins (46–49), and whereas inhibition of CatSper abolished the motility decrease in Ca^2+^-free buffer, it was accelerated about 4-fold upon progesterone activation of the channel (Figure 1A). We surmised that the action of low [Ca^2+^]_o_ on human sperm motility can be harnessed to assess the activity of CatSper by an end-point motility test.

As a proof-of-concept, we diluted the ejaculate from donors with HTF^+^ (Buffer A) and HTF^0Ca^ containing progesterone (Buffer B). After 15 minutes, the fractions of motile sperm in A and B were determined and a CatSper-Activity-Index (CAI) was calculated (see Methods), which ranged from 70–100; however, when Buffer B was fortified with RU1968, the CAI decreased to under 40 (Figure 1B), reflecting that inhibition of CatSper preserves sperm motility in Buffer B. These results supported the notion that the activity of CatSper in human sperm can be assessed by this motility-based test, which we refer to as the CatSper-Activity-Test.

### The CatSper-Activity-Test identifies patients with defective CatSper function.

**Figure 2:**
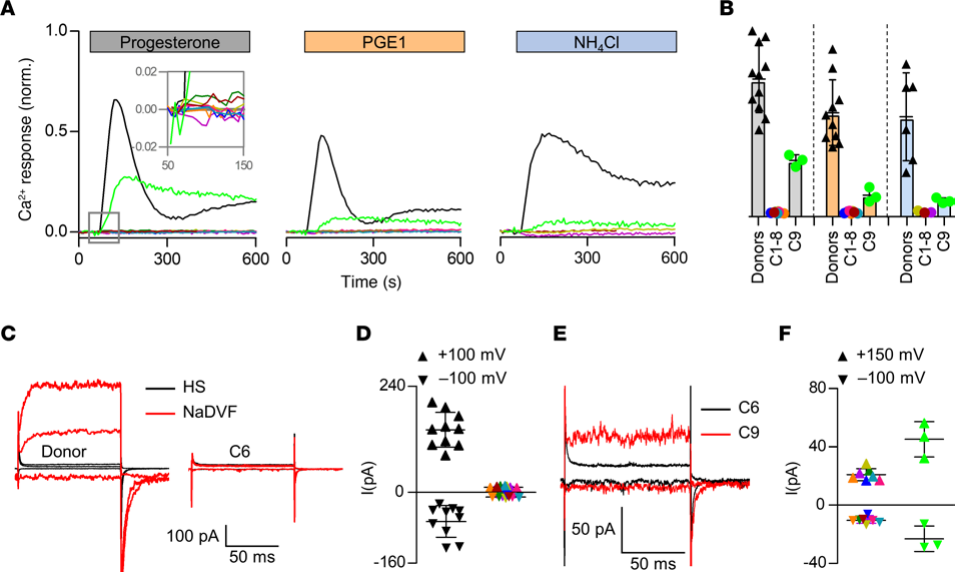
Ca^2+^ signals and membrane currents in sperm from patients with impaired or loss of CatSper function. (**A**) Representative Ca^2+^ signals in sperm from donors and patients C1–C9 (color coded) evoked by progesterone (3 μM), PGE1 (3 μM), or NH_4_Cl (30 mM) relative to the maximal signal amplitude evoked by ionomycin (3 μM) (set to 1). (**B**) Mean (± SD) maximal signal amplitude evoked by progesterone (gray; donors *n* = 11, C1-C8 *n* = 1, C9 *n* = 3), PGE1 (orange; donors *n* = 10, C1,2,4-8 *n* = 1, C9 *n* = 3), or NH_4_Cl (blue; donors *n* = 6, C1,2,8 *n* = 1, C9 *n* = 3) relative to that evoked by ionomycin (set to 1). (**C**) Representative whole-cell currents recorded from a sperm cell of a donor and patient C6 in extracellular solution containing Mg^2+^ and Ca^2+^ (HS) and in Na^+^-based divalent-free solution (NaDVF), evoked by stepping the membrane voltage to –100mV, +100 mV, and +150 mV from a holding potential of –80 mV. (**D**) Steady-state current amplitudes (NaDVF) at +100 mV and –100 mV in sperm from donors (black, *n* = 10) and patients C1–C8 (color coded, *n* = 1). (**E**) Representative whole-cell currents recorded from patients C6 (black) and C9 (red) in NaDVF, evoked by stepping the membrane voltage from to –100mV, +100 mV, and +150 mV from a holding potential of –80 mV. (**F**) Steady-state current amplitudes at +150 mV and –100 mV in NaDVF in sperm from patients C1–C7 and C1–C8, respectively, (color coded; *n* = 1) as shown in **C** (consider the scales of Y-axes) compared to patient C9 (light green, *n* = 3). Data on Ca^2+^ responses and membrane currents in sperm from patients C1–C5 comprise data from ref. 44, reporting on these patients for the first time, combined with data from additional experiments.

We next performed the CatSper-Activity-Test over a period of 3 years on 2,286 men undergoing semen analysis. For more than 99% of the men, the CAI was over 40 (Figure 1C), which we regarded as indicative of normal CatSper function; prototypical CatSper-mediated Ca^2+^ signals recorded in sperm from a representative sample of 48 of those men supported this assumption. In 16 men subjected to the CatSper-Activity-Test, the CAI was, however, similar to that in donors upon inhibition of CatSper, i.e., less-than or equal-to 40, indicative of defective CatSper function (Figure 1C). Of these men, 7 were, however, false positives, i.e., their sperm also showed prototypical CatSper-mediated Ca^2+^ signals. By contrast, a series of follow-up experiments confirmed that 9 men — referred to as patients C1–C9 (Figure 1C, color-coded circles) — indeed featured defective CatSper function. Sperm isolated from ejaculates of patients C1–C8 lacked the Ca^2+^ signals evoked by progesterone-, prostaglandin E1-, or alkaline-activation of CatSper in sperm populations (Figure 2, A and B, colored traces and circles); the Ca^2+^ signal evoked by the Ca^2+^ ionophore ionomycin was preserved. For patients C1 and C2, the lack of CatSper-mediated Ca^2+^ signals was validated on the single-cell level (Supplemental Figure 1). Electrophysiological recordings revealed that sperm from patients C1–C8 also lacked CatSper-mediated membrane currents (Figure 2, C and D). Furthermore, in sperm from patient C9 (Figure 1C, light green circle), CatSper-mediated Ca^2+^ signals (Figure 2, A and B, light green traces and circles, respectively) were about 5- to 10-fold reduced in amplitude and featured an atypical mono- rather than biphasic waveform (Figure 2A); CatSper-mediated membrane currents were also largely, but not entirely, abolished (Figure 2, E and F). Thus, whereas sperm from patients C1–C8 lacked functional CatSper, patient C9 featured a severely impaired CatSper function. These results demonstrate that a fraction of men undergoing a fertility workup suffer from defective CatSper function, which can be identified by the CatSper-Activity-Test.

Of note, we successively optimized the CatSper-Activity-Test during the course of screening to minimize and, finally, avoid false positives (Supplemental Figure 2). Moreover, additional experiments using ejaculates from donors and patients lacking functional CatSper as a tool indicate that the test distinguishes best between normal versus loss of CatSper function when the fraction of motile sperm is determined 60 minutes after dilution in the test buffers. However, additional studies are required to determine the test’s sensitivity and specificity using these optimized conditions.

### Defective CatSper function is linked to germline mutations in CATSPER genes.

**Figure 3:**
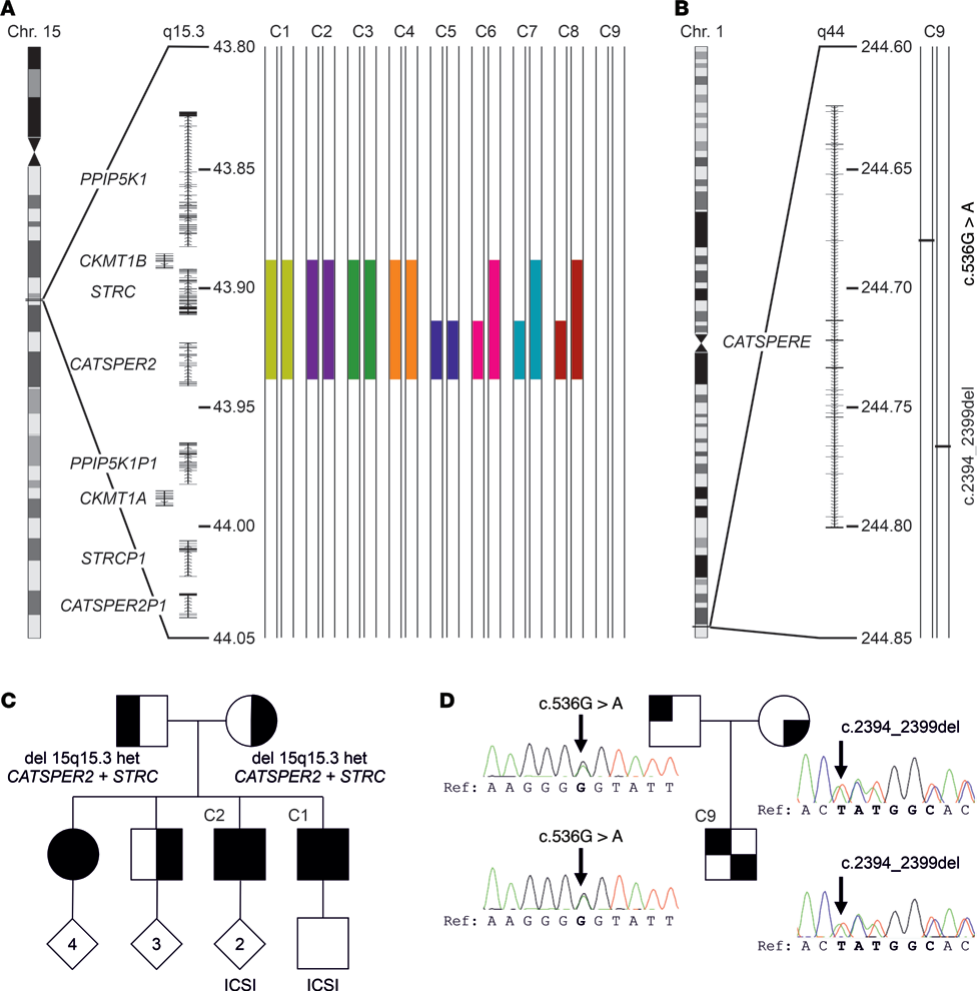
Genetic aberrations identified in patients with impaired or loss of CatSper function. (**A**) Schematic depiction of chromosome 15, magnified region q15.3 including genes and the identified deletions (filled color-coded bars) in patients C1–C8, but not patient C9. All positions according to hg19/GRCh37. (**B**) Schematic depiction of chromosome 1, magnified region q44 and compound-heterozygous variants (see panel **D**) (c.536G>A and c.2394_2399del) of *CATSPERE* (NM_001130957.2) identified in patient C9. (**C**) Family pedigree of patients C1 and C2. Their sister is also homozygous for the deletion at 15q15.3, whereas their mother, father, and third brother are heterozygous carriers. (**D**) Family pedigree of patient C9, demonstrating that the father and mother are carriers of the missense variant (c.536G>A) and in-frame deletion (c.2394_2399del), respectively. Of note, in ref. 44, we previously showed array-CGH data from patients C1–C5, reporting on the deletion of *CATSPER2* in these patients for the first time.

Genetic workup revealed that patients C1–C8 shared a homozygous deletion of the *CATSPER2* gene on chromosome 15 (Figure 3A, Supplemental Figure 3, and Supplemental Table 2). In patients C1–C5, the deletions were similar on both homologous chromosomes and involved, except for patient C5, the homozygous deletion of the *CKMT1B* and *STRC* genes (Figure 3A and Supplemental Figure 3). In patients C6–C8, the homologous chromosomes exhibited distinct deletions, resulting in heterozygous deletion of *STRC* and *CKMT1B* (Figure 3A and Supplemental Figure 3). Patients C1 and C2 are brothers; their parents as well as another brother are heterozygous carriers of the deletion, whereas their sister is homozygous (Figure 3C).

Patient C9 carried the compound-heterozygous nucleotide variants c.536G>A and c.2394_2399del in the *CATSPERE* gene (NM_001130957.2) on chromosome 1 (Figure 3B), inherited from the father and mother, respectively (Figure 3D). The, according to gnomAD, novel c.536G>A variant causes a potentially deleterious amino acid substitution p.(Gly179Glu) (CADD score = 25.7). The variant c.2394_2399del causes an in-frame deletion of 2 amino acids p.Met799_Ala800del, features an allele frequency of 0.001183 (gnomAD), and has already been described in association with CatSper-related male infertility (40, 41).

In summary, homozygous deletion of *CATSPER2* (*CATSPER2^–/–^*) underlies the loss of CatSper function in patients C1–C8. The severely impaired CatSper function in patient C9 is almost certainly due to the variants in *CATSPERE*.

### CATSPER2^–/–^ patients are normozoospermic, but infertile, and require intracytoplasmic sperm injection to father a child.

Next, we assessed the clinical phenotype of the patients. Patients C1–C7 were normozoospermic, i.e., sperm number, motility, and morphology were within reference limits (Table 1 and Supplemental Video 1), demonstrating that deletion of *CATSPER2* and loss of CatSper function do not affect spermatogenesis (36, 40, 42). In patients C8 and C9, sperm number and motility were also within reference limits, but both featured mild teratozoospermia.

**Figure 4:**
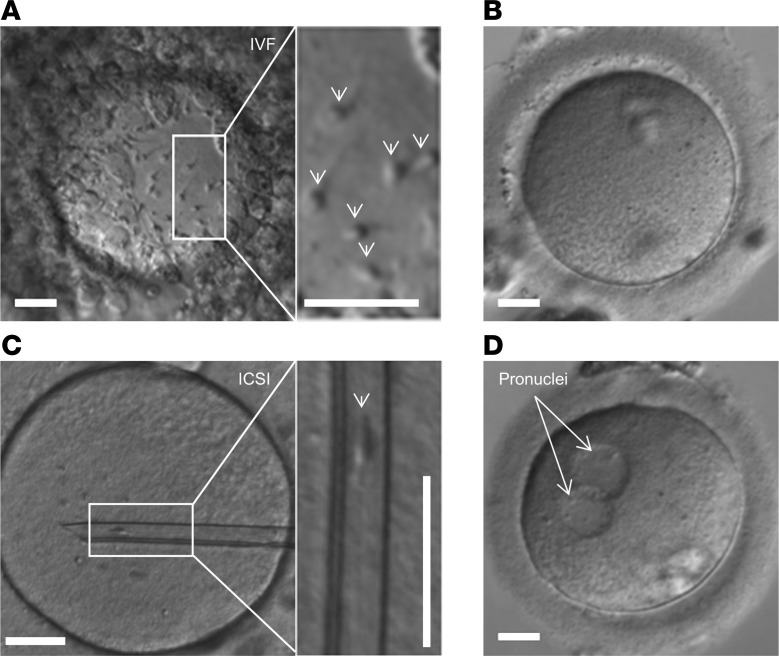
Microscopic documentation of medically assisted reproduction with *CATSPER2^–/–^* sperm. (**A**) Representative micrograph of an oocyte with *CATSPER2^–/–^* sperm from patient C5 attached to the zona pellucida (indicated by arrows in the inset), taken after overnight incubation of sperm and oocyte for in vitro fertilization. (**B**) Representative image of 1 of the 5 oocytes subjected to IVF, none of which was fertilized (total fertilization failure). (**C**) Representative micrograph of an oocyte with an inserted glass pipette containing a *CATSPER2^–/–^* sperm cell from patient C5 (indicated in the inset) for ICSI. (**D**) 9 out of the 15 eggs injected with *CATSPER2^–/–^* sperm developed 2 pronuclei (representative example) indicating fertilization. Scale bars: 30 μm.

Patients C1–C6 and C8 had never conceived a child naturally and presented with unexplained primary couple infertility. Moreover, in these patients, not only natural conception, but also MAR via ovulation induction (OI), intrauterine insemination (IUI), and/or IVF failed (Table 2 and Supplemental Table 3). The 3 normozoospermic patients with proven (40, 42) or presumed (36) loss of CatSper function described before also failed to fertilize upon IVF. Importantly, for patient C5, an IVF/ICSI attempt was documented by video microscopy, providing unprecedented insights. Ten minutes after joining the gametes, sperm were already found bound to the oocytes’ surfaces, which, after 24 hours, were enveloped by sperm (Figure 4A and Supplemental Video 2). However, none of the 5 oocytes subjected to IVF were fertilized (Figure 4B). This total fertilization failure indicates that the sperm failed to penetrate the zona pellucida, thus matching the phenotype of mouse sperm lacking CatSper in IVF (5). Supporting this notion, ICSI performed on 15 oocytes in parallel to the IVF yielded 9 fertilized oocytes (Figure 4, C and D), corresponding to a 60% fertilization rate. Similarly, in patients C1–C4, C6, and C8, fertilization was only achieved by ICSI, which resulted in live births (Table 2 and Supplemental Table 3). Importantly, patients C7 and C9 were childless, but did not present due to suspected infertility; the identification of the defective CatSper function and underlying genetic aberrations came as incidental findings. Altogether, these results demonstrate that loss of CatSper function causes, according to standard fertility workup, unexplained infertility involving OI, IUI, and IVF failure.

Stratification of the study participants (Supplemental Figure 4) revealed that 1.2% of normozoospermic men presenting with couple infertility featured defective CatSper function. Assuming that in half of the infertile couples including a normozoospermic man, the infertility is rather due to a female factor, we estimated a prevalence of 2.3% for CatSper-related male-factor infertility among couples presenting with unexplained infertility (Supplemental Figure 4).

We further examined whether patients lacking *CATSPER2* and *STRC* (*STRC^–/–^*) exhibited sensorineural deafness. Audiometry, indeed, revealed a mild-to-moderate hearing impairment in patients C1–C4 (Supplemental Figure 5), confirming that they have the deafness-infertility syndrome. The cause of their hearing impairment was not known to the patients before. This is not surprising. Although it is well-known that variants in *STRC* represent the second most common genetic aberration causing mild-to-moderate hearing loss, with a prevalence of approximately 4% (39), the gene is still not routinely assessed in the genetic workup of hearing impairment. Therefore, deletions of *STRC* and, thus, also the deafness-infertility syndrome are largely underdiagnosed. Expectedly, in patient C5 with unaffected *STRC* and patient C7 with a heterozygous *STRC* deletion (*STRC*^+/–^), the audiogram was within normal limits (Supplemental Figure 5). For *STRC^+/–^* patients C6 and C8, we did not obtain an audiogram, but neither patient reported impaired hearing.

### Loss of CatSper function affects sperm hyperactivation and migration in viscous media.

**Figure 5:**
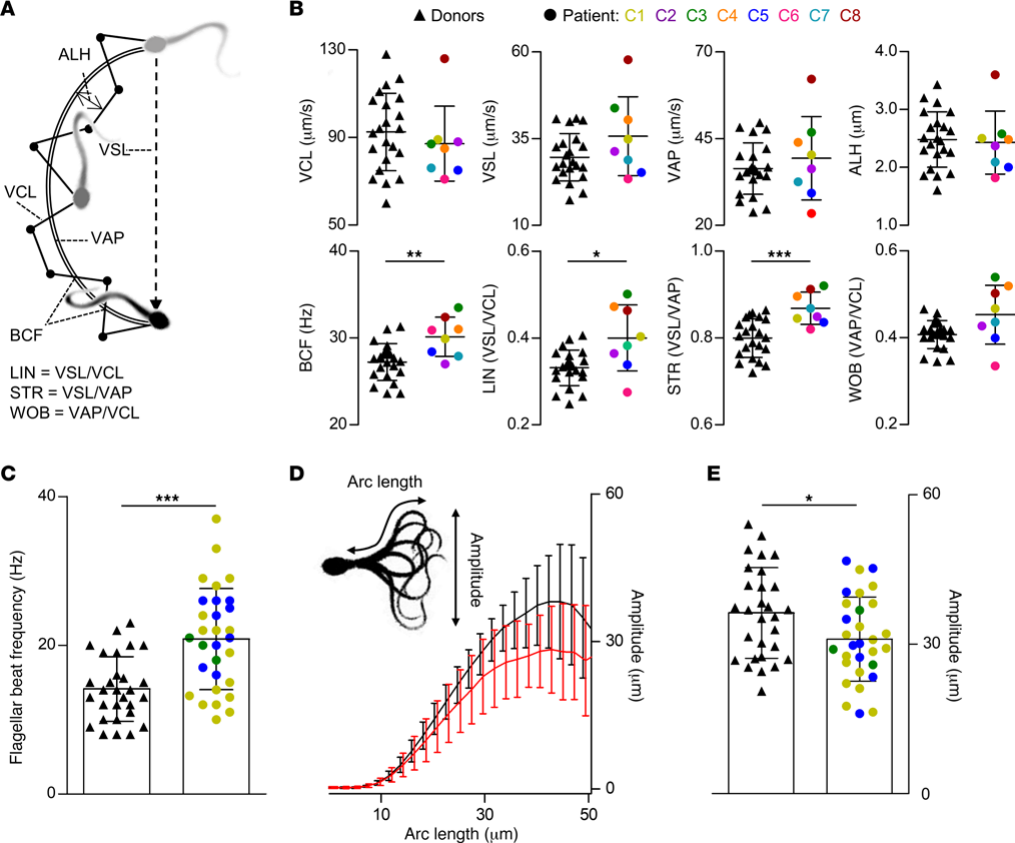
Analysis of basal motility and flagellar beat of control and *CATSPER2^–/–^* sperm. (**A**) Illustration of swimming path and kinematic parameters of a sperm cell determined by CASA. Curvilinear velocity (VCL) represents the frame-to-frame track of the sperm head, from which the average-path velocity (VAP) is calculated. The beat-cross frequency (BCF) is the frequency at which the sperm track crosses the VAP path. The amplitude of lateral head displacement (ALH) is the average deviation of the head from the VAP path. The straight-line velocity (VSL) is derived by charting a direct path between the first and last head position in the image sequence. The linearity (LIN), straightness (STR), and wobble (WOB) are indicators for the linearity of the path trajectory. (**B**) Scatter plots (mean ± SD) of kinematic parameters of control (black triangles, *n* = 22) and *CATSPER2^–/–^* sperm from patients C1–C8 (color-coded circles, *n* = 1). (**C**) Scatter plots (mean ± SD) of the beat frequency of single head-tethered control sperm from donors (black triangles, *n* = 29 from 6 experiments) and patients with *CATSPER2^–/–^* (color-coded circles, *n* = 30 from 3 experiments). (**D**) Maximal beat amplitude (mean ± SD) along the arc length of the flagellum of the head-tethered control (black, *n* = 29) and *CATSPER2^–/–^* (red, *n* = 30) sperm analyzed in **C**. (**E**) Scatter plots (mean ± SD) of the maximal beat amplitude of the control (black triangles, *n* = 29) and *CATSPER2^–/–^* sperm (color-coded circles, *n* = 30) reported on in **C** and **D**. *P < 0.05, **P < 0.01, ***P < 0.001, unpaired *t* test with Welch’s correction.

Next, we set out to unravel the pathomechanism underlying the infertility of *CATSPER2*^–/–^ patients and OI, IUI, and IVF failure, which almost certainly rests on the failure of sperm to penetrate the egg coat. To this end, we investigated the motility of their sperm, referred to as *CATSPER2*^–/–^ sperm, in a population and on the single cell level. First, we diluted the ejaculate of donors, which were used as control samples, and patients with HTF^+^ and quantified the basal sperm-motility parameters in population by computer-assisted sperm analysis (CASA) (Figure 5A). The individual motility parameters were largely similar in *CATSPER2^–/–^* and control sperm (Figure 5B), rendering loss of CatSper function undetectable by CASA. However, when averaged over all donors and patients, beat-cross frequency (BCF; reflecting the beat frequency) and indicators of the linearity of the swimming path (i.e., linearity [LIN] and straightness [STR]) were slightly enhanced in *CATSPER2^–/–^* sperm (Figure 5B). Thus, sperm lacking functional CatSper tend to swim somewhat straighter and with slightly higher beat frequency. Next, we isolated, via swim-up, noncapacitated, motile sperm from the ejaculate of donors and patients with *CATSPER2^–/–^*, tethered their head to the surface of a recording chamber, and analyzed their flagellar beat using the SpermQ software (50) (Figure 5, C–E). Compared with control sperm, *CATSPER2^–/–^* sperm beat with slightly higher frequency and reduced amplitude (Figure 5, C–E), matching the outcome of CASA. These minor anomalies, however, do not explain the infertility and IUI/IVF failure, but rather demonstrate that CatSper does not play a critical role in the control of basal motility features (36, 40, 42). Supporting this notion, in a previous study using motile sperm isolated from the ejaculate of some of the patients with *CATSPER2^–/–^* characterized in detail herein, we showed that loss of CatSper function does not affect rotational motion and rheotaxis either (44).

**Figure 6:**
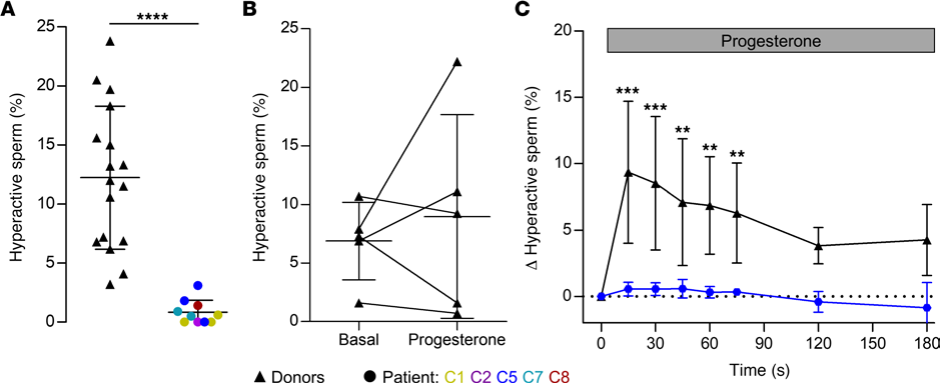
Capacitation- and progesterone-induced hyperactivation of control and *CATSPER2^–/–^* sperm. (**A**) Scatter plot (mean ± SD) of the fraction of hyperactivated control sperm from donors (black triangles, *n* = 17) and *CATSPER2^–/–^* patients (color-coded circles, *n* = 10 experiments with sperm from patients C1, C2, C5, C7, and C8) upon incubation under capacitating conditions. ****P < 0.0001, unpaired *t* test with Welch´s correction. (**B**) Paired plots of the fraction of hyperactivated control sperm before (basal) and after treatment (5 minutes) with progesterone (5 μM) determined by CASA (*n* = 5). (**C**) Change of the fraction (mean ± SD) of hyperactivated control (black, *n* = 5) and *CATSPER2^–/–^* sperm (blue, *n* = 3, i.e., 3 experiments with sperm from patient C5) evoked by mixing with progesterone (gray bar), corrected for the fraction of hyperactivated sperm determined after mixing with HTF^++^ alone (set to 0 at *t* = 0 seconds), and determined by a custom kinetic CASA technique. Experiments were performed with sperm incubated under capacitating conditions. **P < 0.01, ***P < 0.001, ANOVA with Dunnett’s multiple comparison test versus the respective controls (*t* = 0 s).

Next, we studied whether *CATSPER2*^–/–^ sperm are able to undergo hyperactivation; in mice, hyperactivated sperm motility initiated by CatSper is required to penetrate the egg coat and, thus, for fertilization in vivo and in vitro. However, the role of CatSper in human sperm hyperactivation has remained controversial (9, 10). We first determined whether spontaneous hyperactivation that develops during capacitation — a maturation process of sperm inside the female genital tract — is impaired or even abolished in *CATSPER2^–/–^* sperm. To this end, we incubated motile sperm in capacitating HTF buffer (HTF^++^) and determined the fraction of hyperactive sperm by CASA. In donors, the fraction of hyperactive sperm was 12.2% ± 6.1% (*n* = 17), whereas it was only 0.8% ± 1.0% (*n* = 10) in patients with *CATSPER2^–/–^* (Figure 6A), demonstrating that loss of CatSper function abolished spontaneous capacitation-induced hyperactivation (see also Supplemental Figure 6).

We next studied the action of progesterone on the swimming behavior and flagellar beat of capacitated control and *CATSPER2^–/–^* sperm using microfluidics- and optochemistry-aided motility and flagellar-beat analyses. In fact, whether progesterone evokes hyperactivation and whether this can be detected by CASA have been long-standing controversial issues (10, 51). Indeed, when determined 5 minutes after stimulation, progesterone did not significantly increase the fraction of hyperactive sperm (Figure 6B). Considering the transience of progesterone-induced Ca^2+^ signals (see Figure 2A), we surmised that motility responses might also be rather transient. Therefore, we combined CASA with microfluidics to study changes in sperm motility in a time-resolved fashion (see also Supplemental Figure 7). Mixing of control sperm with progesterone evoked a rapid, transient rise in hyperactivation. Within 15 seconds, the fraction of hyperactive sperm increased by 9.4% ± 5.3% (*n* = 5) and then declined again, settling within 120 seconds on a slightly elevated level that was, however, not significantly different from levels observed upon mixing with HTF^++^ alone (Figure 6C). This time course indeed resembles that of progesterone-induced Ca^2+^ responses (Figure 2A). In *CATSPER2^–/–^* sperm, the progesterone-evoked hyperactivation was abolished (Figure 6C), demonstrating that functional CatSper is required.

**Figure 7:**
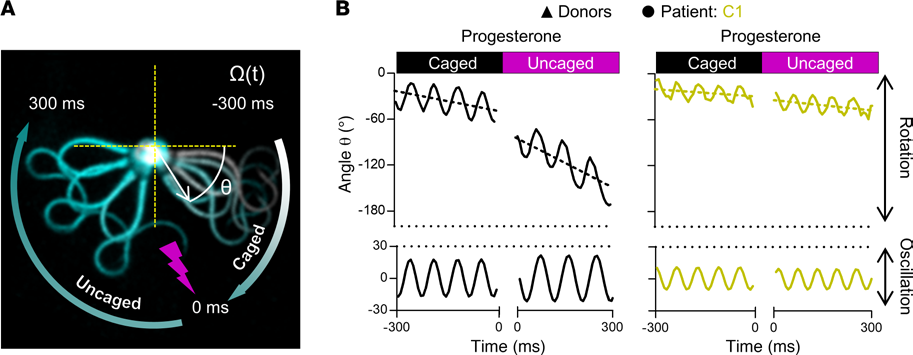
Progesterone-induced changes in flagellar beat frequency and amplitude of control and *CATSPER2^–/–^* sperm. (**A**) Representative time-lapse overlays of 3 beat cycles before (Caged) and after (Uncaged) uncaging progesterone (2 μM) of a pivoting head-tethered capacitated control sperm from a donor. (**B**) Representative change of angle θ over time (solid line, upper panels) with the corresponding slope (dotted line) and fitted sine wave of the oscillation (lower panels) of control (black, left panel) and *CATSPER2^–/–^* sperm (gold, right panel) from patient C1 before and after uncaging progesterone. The corresponding frequency and amplitude of the flagellar beat were derived from the fitted sine wave, and the rotation velocity, Ω (°·s^–1^) were derived by the slope of the change of θ over time. The mean values (± SD) for 9 sperm from donors and patient C1 are provided in Supplemental Figure 8.

To scrutinize this finding by an independent technique, we studied the action of progesterone on the flagellar beat of individual capacitated head-tethered sperm. To this end, we bathed the sperm in HTF^++^ containing caged progesterone (45, 52) and analyzed their motility and flagellar beat before and after uncaging progesterone with a brief UV flash. In general, for head-tethered sperm, the flagellar beat and its inherent asymmetry resulted in an oscillatory rotational motion around the attachment point (Figure 7, A and B) (53, 54). We determined the rotation velocity as well as frequency and amplitude of the oscillation (Figure 7, A and B) in control and *CATSPER2^–/–^* sperm before and after uncaging progesterone (see also Supplemental Videos 3 and 4). Before uncaging progesterone, *CATSPER2^–/–^* sperm featured a slight but insignificant enhanced beat frequency and reduced beat amplitude compared with control sperm, whereas the rotation velocity was similar (Figure 7B and Supplemental Figure 8). In control sperm, uncaging progesterone decreased the beat frequency by 3.1 ± 1.5 Hz (*n* = 9), whereas the beat amplitude and rotation velocity increased by 4.9° ± 7.4° and 95° ± 69°∙s^–1^ (*n* = 9), respectively, suggesting a switch to hyperactive motility (Figure 7B, Supplemental Figure 8, and Supplemental Video 3). In *CATSPER2^–/–^* sperm, progesterone did not affect these motility parameters (Figure 7B, Supplemental Figure 8, and Supplemental Video 4).

**Figure 8:**
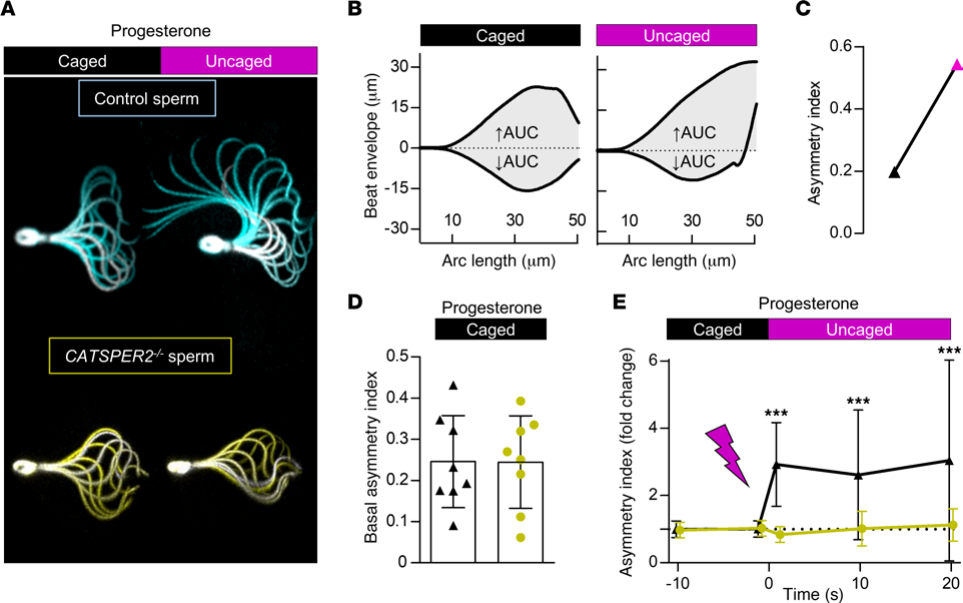
Progesterone-induced changes in flagellar beat asymmetry of control and *CATSPER2^–/–^* sperm. (**A**) Representative overlays of a single beat cycle of a control (cyan) and a *CATSPER2^–/–^* (gold) sperm before (caged) and after uncaging (uncaged) of progesterone (2 μM). (**B**) AUC outlined by the beat envelope above (↑AUC) and below (↓AUC) the head-midpiece axis of a control sperm before and after uncaging progesterone used to derive an asymmetry index (see Materials). (**C**) Paired plot depicting the corresponding asymmetry index of the flagellar beat of the control sperm shown in **A** and **B**. (**D**) The asymmetry index (mean ± SD) of the basal flagellar beat of control (black, 2 donors *n* = 8) and *CATSPER2^–/–^* sperm (gold, patient C1 *n* = 8) before uncaging of progesterone. (**E**) The change in the asymmetry index (mean ± SD) relative to the asymmetry index immediately before (set to 0 at the mean of *t* = –10 seconds and 0 seconds) and after uncaging of progesterone of control (black triangles, 2 donors *n* = 8) and *CATSPER2^–/–^* sperm (gold circles, patient C1 *n* = 8). ***P < 0.001, ANOVA with Dunnett’s multiple comparison test versus the respective controls (*t* = 0 s).

To determine whether progesterone affects also the asymmetry of the flagellar beat, we superimposed images of individual head-tethered sperm recorded during a beat cycle and analyzed these quasi stop-motion images (Figure 8A). Before uncaging, the flagellar beat was largely symmetric in both control (Figure 8A, cyan) and *CATSPER2^–/–^* (Figure 8A, gold) sperm. In control, but not in *CATSPER2^–/–^* sperm, uncaging progesterone induced an increase in beat amplitude and a highly asymmetrical, whip-like beating pattern, which are hallmarks of hyperactive motility (Figure 8A). The changes in beat asymmetry were quantified by determining an asymmetry index (Figure 8, B and C); asymmetry-index values of 0 and 1 indicate perfect beat symmetry and maximal asymmetry, respectively. Prior to uncaging progesterone, the asymmetry index was similarly low in control and *CATSPER2^–/–^* sperm (0.25 ± 0.11 versus 0.24 ± 0.11, *n* = 8) (Figure 8D). Progesterone increased the asymmetry index in control sperm by approximately 2.5-fold, whereas, in *CATSPER2^–/–^* sperm, if there was any change at all, the asymmetry index slightly decreased (Figure 8E). Altogether, the motility and flagellar-beat analyses show that loss of CatSper function abolishes both spontaneous and progesterone-induced hyperactivation.

**Figure 9:**
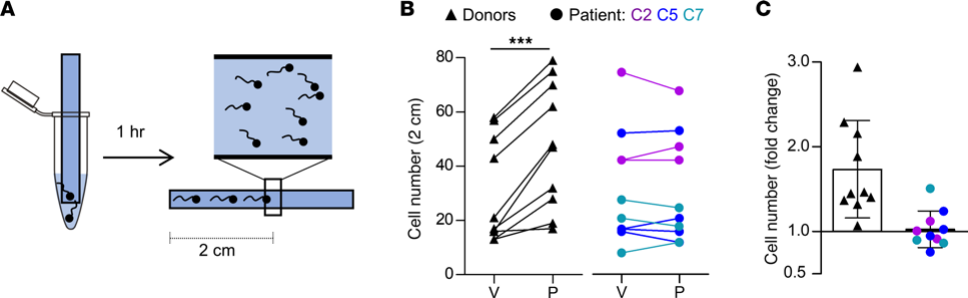
Viscous-media penetration of control and *CATSPER2^–/–^* sperm. (**A**) Experimental layout of the modified Kremer’s sperm-mucus penetration test. Glass capillaries filled with methyl cellulose solution in HTF^++^ fortified with either DMSO (vehicle) or progesterone (3 μM) are placed in tubes containing capacitated sperm in HTF^++^ fortified correspondingly with DMSO or progesterone. After 1 hour, the number of sperm reaching the 2-cm mark were counted. (**B**) Paired plots of the number of control (black triangles, *n* = 10) and *CATSPER2^–/–^* sperm (color-coded circles, *n* = 10) from 4 independent experiments with sperm from patients C2, C5, C7) at 2 cm in the presence of the vehicle (V) or progesterone (P). (**C**) Fold change in the number of control (black triangles, *n* =10) and *CATSPER2^–/–^* sperm (color-coded circles, *n* = 10) at 2 cm in the presence of progesterone, relative to the vehicle (set to 1); ***P < 0.001, paired *t* test.

We also studied the migration of sperm into a viscous medium using a modified Kremer’s sperm-mucus penetration test. An open glass capillary containing HTF^++^ fortified with methylcellulose was partially submersed in a suspension of sperm incubated under capacitating conditions in HTF^++^ (Figure 9A). In the absence of progesterone, numbers of control and *CATSPER2^–/–^* sperm penetrating the viscous medium were similar (Figure 9B), demonstrating that penetration into viscous medium, per se, does not require CatSper (40, 42). In the presence of progesterone, the number of control sperm was enhanced by about 1.7-fold (Figure 9, B and C), confirming that progesterone facilitates the migration into viscous medium (40, 42, 55–57). This progesterone action was abolished in *CATSPER2^–/–^* sperm (Figure 9, B and C), demonstrating that it requires functional CatSper (40, 42).

**Figure 10:**
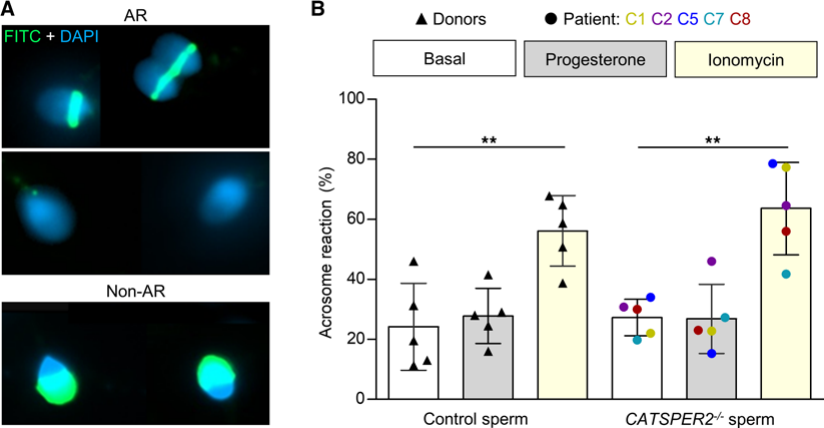
Acrosome reaction of control and *CATSPER2^–/–^* sperm. (**A**) Representative fluorescence images of capacitated nonacrosome reacted (non-AR) and acrosome reacted (AR) control sperm from donors stained with DAPI (blue) and FITC-labelled peanut agglutinin (PNA) lectin. (**B**) Scatter plot of the fraction (mean ± SD) of acrosome-reacted control (black triangles, *n* = 5) and *CATSPER2^–/–^* sperm (color coded, *n* = 5) after incubation in HTF^++^ (basal; white), progesterone (5 μM; gray), or ionomycin (5 μM; yellow). Experiments were performed with sperm incubated under capacitating conditions. **P < 0.01, ANOVA with Bonferroni’s multiple comparisons test versus the respective basal values.

For fertilization, human sperm might also need to undergo acrosomal exocytosis (58). We studied acrosomal exocytosis using FITC-labeled pisum sativum agglutinin (FITC-PSA) as a marker (Figure 10A). Spontaneous and ionomycin-induced acrosomal exocytosis were similar in control and *CATSPER2^–/–^* sperm (Figure 10B), confirming that it is not affected by loss of CatSper function (ref. 42; see also ref. 36 for contrasting information). To elucidate the role of Ca^2+^ influx via CatSper, we studied the action of progesterone, which, in most (42, 45, 56, 57, 59–62) but not all (63, 64), previous studies sufficed as a stimulus for acrosomal exocytosis and was abolished in sperm lacking functional CatSper (42). Under our experimental conditions, for unknown reasons, the fraction of acrosome reacted control sperm was not significantly enhanced by progesterone (Figure 10B), which precluded scrutinizing the role of Ca^2+^-influx via CatSper for acrosomal exocytosis.

## Discussion

More than 2 decades ago, CatSper and its essential role in sperm function and male fertility in mice were discovered (5). Since then, the role of CatSper in spermatogenesis, sperm function, and male fertility in humans has remained ill defined and controversial (9, 10). To clarify these long-standing issues, we developed the CatSper-Activity-Test, screened approximately 2,300 individuals, identified a group of men with defective CatSper function, assessed their reproductive phenotype, and investigated the behavior of human sperm lacking functional CatSper. This comprehensive approach unveiled that CatSper is not required for sperm production in men. In fact, defective CatSper function is undetectable by the current fertility workup, but it causes failure of MAR via OI, IUI, and IVF (36, 40, 42) — techniques of primary choice for couples with unexplained infertility.

We propose that defective CatSper function leads to a similar infertility phenotype in mice and humans. Using several complementary techniques, we show that human sperm lacking functional CatSper fail to hyperactivate spontaneously and upon hormone stimulation. Moreover, we provide experimental evidence that the sperm fail to penetrate the zona pellucida. This indicates that sperm hyperactivation initiated by CatSper is required for human fertilization in vivo and in vitro, explaining the infertility of men lacking functional CatSper, failure of OI, IUI,and IVF, as well as the need for ICSI.

Yet, loss of CatSper function also affects migration in viscous medium, which could prevent the sperm to even reach the site of fertilization in vivo. Hence, human sperm might also be instructed by physicochemical cues to locate the oocyte by rheotaxis, thermotaxis, chemotaxis, or a combination thereof (3). CatSper is not required for rheotaxis (44), but the role of CatSper in human sperm chemotaxis and thermotaxis remains to be elucidated. Thus, studies mimicking the chemical, topographical, and hydrodynamic landscapes of the female genital tract in vitro (30, 65, 66) are required to gain insights into the role of CatSper for sperm navigation in vivo. Future studies are also required to understand the role of Ca^2+^ influx via CatSper for acrosomal exocytosis using, for example, physiological combinations of ligands and/or intracellular alkalization, which is more efficacious than progesterone alone in stimulating Ca^2+^ influx via CatSper (64, 67) and acrosomal exocytosis (62, 64). Finally, whether impaired CatSper function, as seen in patient C9, also affects sperm function and male fertility remains to be determined; in mice, impaired rather than total loss of CatSper function also affects hyperactivation, resulting in sub- or infertile male mice (17, 18, 20). This requires the identification of more patients with impaired, rather than loss of, CatSper function. With this identification, we can unravel if they can naturally conceive. This requires us to identify more patients with impaired, rather than loss of, CatSper function and performance of an in-depth analysis of their fertility phenotype the function of their sperm. This might reveal the critical level of CatSper activity for sperm hyperactivation and thus natural conception, IUI, and/or IVF.

We further show that homozygous deletion of *CATSPER2* is the main cause affecting CatSper function. In only half of the patients with *CATSPER2^–/–^*, *STRC* was also deleted, representing the patients with hearing loss. Our findings match with population genetics; among the (likely) pathogenic variants identified in genes encoding CatSper subunits, deletion of *CATSPER2* is by far the most common, with an allele frequency of about 1% each for deletion of *CATSPER2* or *CATSPER2* + *STRC* (gnomAD SVs 2.1). The high deletion frequency is most likely due to the architecture of the locus. The *PPIP5K1, CKMT1, STRC*, and *CATSPER2* genes share over 98% sequence homology (32) with their, except for *CKMT1*, pseudogenic counterparts created by segmental duplication (Figure 3). This renders the genes prone to nonallelic homologous recombination (NAHR), which results in sequence deletions, duplications, or inversions (33, 68). Of note, the patients from our study lacking *CATSPER2*, *STRC*, and *CKMT1B* were normozoospermic, demonstrating that neither *CATSPER2*, *STRC*, nor *CKMT1B* are required for spermatogenesis. Moreover, the infertility phenotype of patients with homozygous deletion of *CATSPER2* (patient C5), homozygous deletion of *CATSPER2* combined with heterozygous deletion of *STRC* and *CKMT1B* (patients C6–C8), or homozygous deletion of *CATSPER2*, *STRC*, and *CKMT1B* (patients C1–C4) was indistinguishable. This demonstrates that the infertility phenotype was caused by the homozygous deletion of *CATSPER2* alone rather than by deletion of *STRC* and/or *CKMT1B*.

Further, in sperm from a proven father heterozygous for the deletion of *CATSPER2* (*CATSPER2*^+/–^), CatSper-mediated membrane currents as well as motility parameters were unaffected (Supplemental Figure 9), supporting fully penetrant autosomal-recessive inheritance of *CATSPER2*-related infertility. The c.2394_2399del variant in *CATSPERE* of patient C9 has already been associated with CatSper-related male infertility (40, 41) and features an allele frequency of 0.01%. The c.536G>A variant in *CATSPERE* as well as those previously described for *CATSPER1* and *CATSPER3* are much less frequent or even novel.

Furthermore, from the allele frequencies in the general population, we deduce a prevalence for defective CatSper function due to homozygous deletion of *CATSPER2* of approximately 0.01%, i.e., 1 in 10,000 men are affected. In the group of men enrolled in our study, the prevalence was orders of magnitudes higher (Supplemental Figure 4). This enrichment is to be expected, because we selected for men with fertility disorders. Nevertheless, even among the 500 men that presented for other reasons, we identified 2 with defective CatSper function. Similarly, because of the frequent concomitant deletion of *STRC*, the prevalence for homozygous deletion of *CATSPER2* is 3 orders of magnitude higher in men with mild-to-moderate hearing loss than predicted for the general population (39, 69).

Because CatSper-related male infertility is undetectable by semen analysis, affected couples experience failed OI, IUI, and IVF treatments (Table 2). This is frustrating, time-consuming, expensive, and, most importantly, involves repeated treatment of the woman. Thus, early diagnosis of this sperm channelopathy enables evidence-based selection of the MAR technique, shortening the time toward reproductive success for affected couples, reducing psychological burdens and expenses, and minimizing the medical risk for the woman. We find the CatSper-Activity-Test to be relatively simple and beneficial; it could be performed along with semen analysis to identify patients with defective CatSper function. Importantly, its readout reveals defective CatSper function irrespective of the underlying genetic or nongenetic pathomechanism. In fact, such transfer of basic knowledge into the clinics, improving diagnosis and care of patients, has been lacking in the field. Our study might, thus, serve as a blueprint for future studies unraveling the causes of unexplained infertility and developing novel diagnostic tools.

## Methods

### Reagents

Chemicals were purchased from AppliChem, Carl Roth, Cayman Chemical, Thermo Fisher Scientific, Honeywell Fluka, Sigma-Aldrich, Merck, VWR, and Irvine Scientific.

### Sperm preparation and buffer conditions

Semen samples provided by donors and patients for research were produced by masturbation and ejaculated into plastic containers. Ejaculates were allowed to liquefy at 37°C for 30–60 minutes. Motile sperm were isolated by a swim-up procedure: in a 50-mL tube, aliquots of 0.5 to 1 mL ejaculate were layered under 4 mL of human tubal fluid (HTF) containing (in mM): 97.8 NaCl (Carl Roth), 4.69 KCl (Carl Roth), 0.2 MgSO_4_ (Honeywell Fluka), 0.37 KH_2_PO_4_ (Merck), 2.04 CaCl_2_ (Carl Roth), 0.33 Na-pyruvate (Carl Roth), 21.4 lactic acid (Sigma-Aldrich), 2.78 glucose (Carl Roth), 21 HEPES (Carl Roth), and 4 NaHCO_3_ (Sigma-Aldrich); adjusted between pH 7.3–7.4 with NaOH (AppliChem). Alternatively, the ejaculate was diluted 1:10 with HTF, and sperm, somatic cells, and cell debris were pelleted by centrifugation (700*g*, 20 minutes, 37°C). The sediment was resuspended in the same volume of HTF in 50-mL tubes. Aliquots of 5 mL of this suspension were pelleted in the 50-mL tubes using a centrifuge with a fixed-angle rotor (700*g*, 5 minutes, room temperature). In both cases, motile sperm were allowed to swim up for at least 60 minutes at 37°C, collected with the supernatant, and washed twice with HTF (700*g*, 20 minutes, 37°C). After the second centrifugation, sperm were resuspended in HTF supplemented with 3 mg/mL HSA (Irvine Scientific; dubbed HTF^+^), or in capacitating HTF^++^ containing (in mM): 72.8 NaCl, 4.69 KCl, 0.2 MgSO_4_, 0.37 KH_2_PO_4_, 2.04 CaCl_2_, 0.33 Na-pyruvate, 21.4 lactic acid, 2.78 glucose, 25 NaHCO_3_, and 21 HEPES, pH 7.35 (adjusted with NaOH), and supplemented with 3 mg/mL HSA. The sperm density was determined using a Neubauer chamber and adjusted to 1 × 10^7^ sperm/mL. Sperm were incubated at 37°C under capacitating conditions in HTF^++^ for at least 2 and 3 hours for acrosome-reaction assays and motility analysis, respectively.

For motility experiments designed to study noncapacitated and capacitated sperm side-by-side, an aliquot of the ejaculate was subjected to swim-up and washed in bicarbonate-free HTF (HTF^0BC^) containing (in mM): 97.8 NaCl, 4.69 KCl, 0.2 MgSO_4_, 0.37 KH_2_PO_4_, 2.04 CaCl_2_, 0.33 Na-pyruvate, 21.4 lactic acid, 2.78 glucose, and 21 HEPES, pH 7.35 (adjusted with NaOH) without HSA, representing noncapacitating conditions.

### Analysis of sperm-motility decay in Ca2+-free buffer

Swim-up sperm in HTF^+^ were diluted tenfold in HTF^+^ or Ca^2+^-free HTF (HTF^0Ca^; [Ca^2+^]_o_ < 20 nM), containing (in mM): 91.8 NaCl, 4.69 KCl, 0.2 MgSO4, 0.37 KH_2_PO_4_, 5 EGTA (Carl Roth), 0.33 Na-pyruvate, 21.4 lactic acid, 2.78 glucose, 25 NaHCO_3_, and 21 HEPES, adjusted to pH 7.35 with NaOH, supplemented with 3 mg/mL HSA. The fraction of motile sperm was monitored over time. The time constant, τ, of the motility decay was derived by nonlinear regression analysis using the 1-phase decay equation:
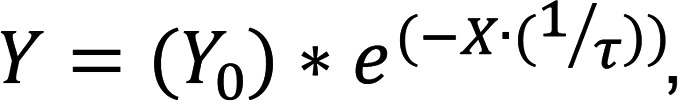


where Y is the fraction of motile cells (%), Y_0_ is the initial fraction of motile cells (constrained to 100%), and x is the time. To study the role of CatSper, the buffer was fortified with progesterone (Cayman Chemical) or RU1968 (45).

### CatSper-Activity-Test

The CatSper-Activity-Test was performed on men undergoing semen analysis in the course of a fertility workup, provided that the ejaculate featured a total sperm count of at least 5 × 10^6^ and a minimum of 10% total motile sperm. We also performed the test on ejaculates from donors. A 20-μL aliquot of the ejaculate was diluted tenfold in HTF^+^ (Buffer A) and HTF^0Ca^ containing 10 μM progesterone (Buffer B) or Buffer B including 5 mM EDTA (Carl Roth). To derive the CatSper-Activity-Index (CAI), the fraction of motile sperm in Buffer A and Buffer B was determined after 15 or 30 minutes and in Buffer B including EDTA after 30 or 60 minutes according to the equation:
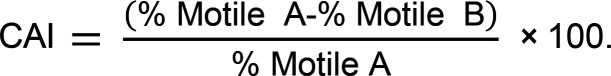


### Measurement of [Ca2+]i changes in sperm populations

Changes in [Ca^2+^]_i_ were measured in swim-up sperm (in HTF^+^) loaded with the Ca^2+^ indicator Fluo-4-AM (Thermo Fisher Scientific) in 384 multi-well plates in a fluorescence plate reader (Fluostar Omega, BMG Labtech) at 30°C as described previously (47, 54, 67). Briefly, sperm were loaded with Fluo-4-AM (5 μM, 20 minutes) at 37°C in the presence of Pluronic F-127 (Sigma-Aldrich; 0.05% w/v). After incubation, excess dye was removed by centrifugation (700*g*, 5 minutes, room temperature). Sperm were resuspended in HTF at a density of 5 × 10^6^ cells/mL, and wells were filled with 54 μL of the suspension; Fluo-4 was excited at 480 nm, and emission was recorded at 520 nm. Changes in fluorescence were converted to changes in ΔF/F_0_ (%), indicating the percentage change in fluorescence (ΔF) with respect to the mean basal fluorescence (F_0_) before application of buffer or stimuli. ΔF/F_0_ (%) changes evoked by progesterone, PGE1 (Cayman Chemical), or NH_4_Cl (Carl Roth) were normalized to the maximal increase in ΔF/F_0_ (%) evoked by ionomycin (Cayman Chemical).

### Single-cell Ca2 fluorimetry

Slides (10-well CELLview slide, Greiner Bio-One) were prepared by coating the wells for 60 minutes at 37°C in a 1:10 aqueous mixture of poly L-lysine (Sigma-Aldrich; 1 mg/mL) and sodium borate buffer (Carl Roth; 1 M, pH 8.5). Wells were washed with HTF^+^ and left to dry. Swim-up sperm in HTF^+^ were loaded with Fluo-4-AM (5 μM, 60 minutes) at 37°C in the presence of Pluronic F-127 (0.05% w/v). After incubation, excess dye was removed by centrifugation (700*g*, 5 minutes, room temperature), and sperm were resuspended in HTF^+^ to a density of 5 × 10^6^ cells/mL, aliquoted (200 μL) into the wells, and incubated at 37°C and 10% CO_2_ for 1 hour to allow sperm to settle. Wells were then washed with HTF^+^ and placed under an inverted microscope (IX73, Olympus) with a condenser (IX2- LWUCD) and a custom-made dark-field filter, housed in an incubator with circulating air heated to 37°C, and equipped with a 20× objective (UPLFLN20XPH, Olympus). Fluorescence was excited by LED (475/35 nm, Thorlabs) and image sequences were recorded at 1 frame per second (fps) for up to an hour using the sCMOS camera (Zyla 4.2 Plus, Andor Technology). After obtaining a baseline, 22 μL of progesterone (3 μM final) or HTF^+^ (control) was added to followed by 24.4 μL of ionomycin (1 μM final). Image sequences were analyzed using ImageJ (NIH) and the change in fluorescence intensity (ΔF/F_0_) of selected cells was normalized to the ionomycin signal.

### Electrophysiology

We recorded from swim-up sperm in the whole-cell configuration as described before (47). Seals between pipette and sperm were formed at the cytoplasmic droplet or neck region in extracellular solution (HS) containing (in mM): 135 NaCl, 5 KCl, 1 MgSO_4_, 2 CaCl_2_, 5 glucose, 1 Na-pyruvate, 10 lactic acid, and 20 HEPES, adjusted to pH 7.4 with NaOH. Monovalent currents were recorded in a sodium-based divalent-free solution (NaDVF) containing (in mM): 140 NaCl, 40 HEPES, and 1 EGTA, adjusted to pH 7.4 with NaOH; the pipette (10–15 MΩ) solution contained (in mM): 130 Cs-aspartate (Merck), 5 CsCl (Carl Roth), 50 HEPES, and 5 EGTA, adjusted to pH 7.3 with CsOH (Sigma-Aldrich). Data were not corrected for liquid junction potentials.

### Genetics analyses

Genomic DNA was extracted via local standard methods from peripheral blood or buccal swabs.

#### Single nucleotide polymorphism array.

Genetic workup started with single nucleotide polymorphism–array (SNP-array) analysis using the Infinium CytoSNP-850K v1.2 BeadChip Kit (Illumina) according to manufacturer’s protocol. The BeadChips were scanned using the NextSeq 550 System (Illumina). Data was analyzed with BlueFuse Multi v4.5 (Illumina).

#### Multiple ligation-dependent probe amplification.

To determine the extent of deletions on chromosome 15 identified in patients C1–C8 via the SNP array, the SALSA MLPA Probemix P461-A1 DIS (MRC Holland) kit was used, which is designed to analyze individuals with sensorineural hearing loss and detect deletions or duplications in the genes *STRC*, *CATSPER2* and *OTOA*. Data was analyzed with Coffalyser.Net analysis software (MRC Holland).

#### Exome sequencing and analysis.

Because patient C9 did not feature a deletion of *CATSPER2*, we sequenced his exome. After enrichment with Twist Bioscience’s Human Core Exome kit, sequencing was conducted on the Illumina NovaSeq 6000 system using the TruSeq SBS Kit v3 - HS (200 cycles). The bioinformatic analyses have been described previously (70). In brief, trimming and alignment of reads (Cutadapt v1.18, BWA Mem v0.7.17) and calling and annotation of variants (GATK toolkit v3.8, Ensembl Variant Effect Predictor v100) were carried out. Exome data were screened focusing on rare (minor allele frequency [MAF] under 1% in the gnomAD database, v2.1.1) biallelic variants predicted to affect protein function (stop-gain, frameshift, and splice site variants as well as missense variants with a Combined Annotation Dependent Depletion [CADD] score of at least 20) in genes encoding CatSper channel subunits (Supplemental Table 1).

#### Sanger sequencing.

Validation of variants in C9 and segregation analysis in family members were performed by Sanger sequencing. For primer sequences, see Supplemental Table 4.

### Standard CASA

Custom motility chambers (depth approximately 60 μm) were prepared by applying 2 pieces of adhesive tape to a glass slide approximately 1 centimeter apart. Samples (30 μL) of diluted ejaculates or swim-up sperm were placed between the tape strips and covered with a coverslip. For basal motility analysis, dark-field images of ejaculates diluted 1:10 in HTF^+^ were recorded with a CMOS camera (UI-3140CP-M-GL R2, iDS GmbH) at 80 fps for 1 second with an upright microscope (BX-40, Olympus) equipped with a heated (37°C) stage (HT 50, Minitüb), a 10× objective (UPLANFL 10X, Olympus), and a 0.5× magnification adapter (U-TV0.5XC-3, Olympus; Hamburg, Germany) (total magnification: 5×). Image stacks were analyzed in ImageJ (NIH) with an open-source CASA program (71, 72) adapted for human sperm.

Hyperactivation of noncapacitated swim-up sperm (1 × 10^6^ cells/mL) prepared in HTF^0BC^ was measured by supplementing HSA (3 mg/mL) only immediately before the experiment to prevent attaching of sperm to the surface of the recording chamber. To study hyperactivation of capacitated swim-up sperm, the sperm suspension was diluted to 1 × 10^6^ cells/mL in HTF^++^ before transferring to the recording chamber. Sperm with a curvilinear velocity (VCL) of at least 150 μm/s, linearity (LIN) of 0.5 or less, and amplitude of lateral head displacement (ALH) of at least 7 μm were classified as hyperactive (73).

### Microfluidic rapid-mixing CASA

Progesterone-evoked motility changes in swim-up sperm were measured with the inverted IX73 microscope and incubator setup used for single-cell Ca^2^ fluorimetry, equipped with the 0.5× magnification adapter and 10× objective used for standard CASA analysis. A microfluidic chamber with 3 inlets (μ-Slide III 3in1, Ibidi) was connected via 3/2-way valves to the following reservoirs: (a) capacitated sperm (5 × 10^6^ cells/mL HTF^++^), (b) HTF^++^, and (c) HTF^++^ containing progesterone (10 μM). The single outlet of the chamber was connected to a syringe pump (World Precision Instruments), which, by setting the 3/2-way valves, pulled fluid from 2 designated reservoirs (e.g., sperm and HTF^++^ [control] or sperm and HTF^++^ containing progesterone) under laminar flow, priming the chamber for mixing. By abruptly reversing the pump, the laminar fluid layers inside the chamber were turbulently mixed. Time-stamped dark-field images were continuously recorded over 180 seconds with the CMOS camera (80 fps) also used for standard CASA. 3 1-second image sequences were extracted from the following time frames for analysis with the ImageJ-based CASA program as described before: 0–15, 16–30, 31–45, 46–60, 61–75, 105–120, and 165–180 seconds. The CASA results of the respective time frames were pooled to calculate the overall percentage of hyperactivated sperm.

### Flagellar-beat analysis of head-tethered sperm

The basal flagellar waveform of swim-up sperm was analyzed using the IX73 microscope, condenser, and dark-field filter setup also used for single-cell Ca^2+^ fluorimetry, equipped with a 20× objective (UPLFLN20XPH, Olympus). Dark-field image sequences (250 fps, 5 seconds) of single spermatozoa were recorded using a sCMOS camera (Zyla 4.2 Plus, Andor Technology) and analyzed in ImageJ using the software tool SpermQ (50) (Version v0.1.6; results are reproducible with online version v0.2.0 of SpermQ, master branch, commit d43332f) https://github.com/hansenjn/SpermQ To measure progesterone-evoked motility responses, sperm suspension was fortified with 2 μM caged-progesterone (52), and sperm were recorded (135 fps) 15 seconds before and after photo-release (2 seconds illumination at 365 nm) of progesterone.

#### Asymmetry index.

The maximal beat amplitude along the arc length of the flagellum above and below the head-midpiece axis of the sperm was measured to determine the beat envelope. The “asymmetry index” was calculated by taking the ratio of the area under the beat envelope, i.e., area under the curve (AUC), generated by the beat envelope above (↑AUC) and below (↓AUC) the head-midpiece axis, respectively, according to the following equation:
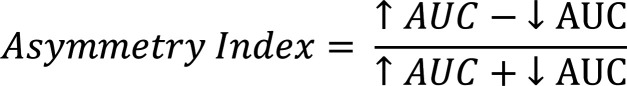


Hence, an asymmetry index of 0 indicates perfect beat symmetry, while a value of 1 indicates complete asymmetry. The asymmetry index was measured before and after the photo release of progesterone from the caged progesterone.

### Viscous media penetration test

Capillary tubes (0.2 × 4.0 mm) (VitroTubes, VitroCom) were filled by capillary action with HTF^++^ supplemented with 1% w/v methyl cellulose (Carl Roth) containing progesterone (3 μM) or vehicle (DMSO; Carl Roth) and capped with sealing wax (Glaswarenfabrik Karl Hecht). Swim-up sperm were capacitated in HTF^++^ in aliquots of 300 μL at 3 × 10^6^ cells/mL. After adding progesterone (3 μM) or vehicle (DMSO), the capillary tubes were placed upright in these sperm suspensions, and placed in an incubator (37 °C, 10% CO_2_). After 1 hour, sperm within a field-of-view corresponding to 600 μm above and below the 2-cm mark inside the capillary were counted, and the fraction of motile sperm in the suspension was determined using the upright BX-40 microscope. The number of cells at the 2-cm mark was normalized to the fraction of motile sperm, correcting for inter-sample variation.

### Acrosome reaction

The evaluation of acrosome reaction was performed based on a slightly modified protocol previously described (45, 60). Briefly, capacitated swim-up sperm in HTF^++^ (2 × 10^6^ cells/mL) were incubated with progesterone (5 μM) or ionomycin (5 μM) for 1 hour at 37 °C, or supplemented with the equivalent volume of HTF^++^ (control). Afterward, sperm were washed by centrifugation (700*g*, 5 minutes, room temperature) and resuspended in hypoosmotic swelling medium (1:10 HTF^++^:ddH_2_O) for 1 hour at 37°C. After another washing, sperm were fixed in 50 μL of ice-cold methanol (VWR), transferred on a slide in 5 aliquots (10 μL per spot), air-dried, and stored at –20 °C. For acrosome staining, the fixed sperm were thawed and incubated with 1 mg/mL FITC-labelled *Arachis hypogaea* agglutinin (PNA) lectin (Sigma-Aldrich) in PBS at 4 °C for 20 minutes in the dark. Slides were washed with PBS and incubated for 10 seconds with 5 mg/mL DAPI (Thermo Fisher Scientific) in ddH_2_O at room temperature, washed again with PBS, and air dried. Images from the slides were taken using an AXIO Observer microscope (Carl Zeiss). For each condition, 200 curled-tail (viable) sperm were considered for analysis. Acrosomal status was assessed manually in a blinded fashion by 2 independent operators using a custom-designed counting aid software. The results of the 2 independent assessments were averaged.

### Statistics

Experiments were performed and analyzed without randomization and blinding, except for the acrosome reaction test. Data are presented as mean ± SD. Statistical analysis was performed using GraphPad Prism 5 (GraphPad Software). If the experiment involved 2 conditions (control and treatment), a 2-tailed paired *t* test was used. Results from donors and patients obtained under the same conditions were also analyzed using 2-tailed unpaired *t* test with Welch’s correction. ANOVA was used for experiments involving 1 parameter measured over time (repeated measures with Dunnett’s multiple comparisons posthoc test) or more than 1 treatment (1-way with Bonferroni’s multiple comparisons posthoc test). A *P* value < 0.05 was considered significant.

### Study approval

The study involved men that underwent semen analysis at the Centre of Reproductive Medicine and Andrology, Münster, Germany, as well as voluntary donors. Data documented in our clinical database *Androbase* (74) were used for patient phenotyping. All participants gave written informed consent according to the protocols approved by the Ethics Committee of the Ärztekammer Westfalen-Lippe and the Medical Faculty Münster (4INie, 2021-402-f-S, and 2010-578-f-S) and the Declaration of Helsinki. Genetic analyses were performed within the Male Reproductive Genomics (MERGE) study (75). The MERGE subject IDs of the *CATSPER2* and *CATSPERE* patients are: C1 = M1, C2 = M1B1, C3 = M1755, C4 = M1782, C5 = M2107, C6 = M2157, C7 = M2108, C8 = M2212, and C9 = M2439.

### Data availability

All the data included in the manuscript is available in the Supporting Data Values file. The SNP-array data on patients C1–C9 and exome-sequencing data of patient C9 has not been deposited in a public repository, because the patients’ consent did not include this. All data including the SNP-array and exome data can, however, be made available by the corresponding author upon request.

## Author contributions

C Brenker, CS, and TS conceived the project. SY, CS, C Brenker, FT, and TS designed the research and interpreted the results. SY, CS, AW, JP, AP, MK, C Biagioni, L Herrmann, BS, AR, TW, L Haalck, DD, C Brenker, and TS performed experiments and analyzed the data. VN, TP, CK, JNH, DW, BR, HMB, SS, and SK contributed to the design of research and/or analysis and interpretation of results. CK, SK, and FT recruited the men enrolled in the study. CK, VN, TP, AR, HMB, SK, and FT performed the genetic and clinical workup and treatment of patients/couples. SY and TS wrote the manuscript. CS, C Brenker, and FT contributed to the writing of the manuscript. Except for DD, who passed away in 2021, all authors revised the manuscript critically for important intellectual content and approved the manuscript.

## Supplementary Material

Supplemental data

Supplemental video 1

Supplemental video 2

Supplemental video 3

Supplemental video 4

Supporting data values

## Figures and Tables

**Table 1:**
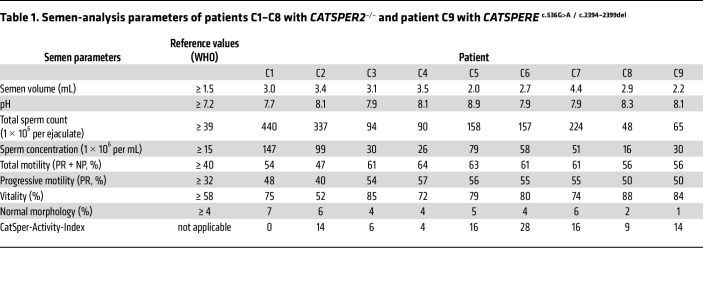
Semen-analysis parameters of patients C1–C8 with *CATSPER2^–/–^* and patient C9 with *CATSPERE* ^c.536G>A^^/^^c.2394–2399del^

**Table 2:**
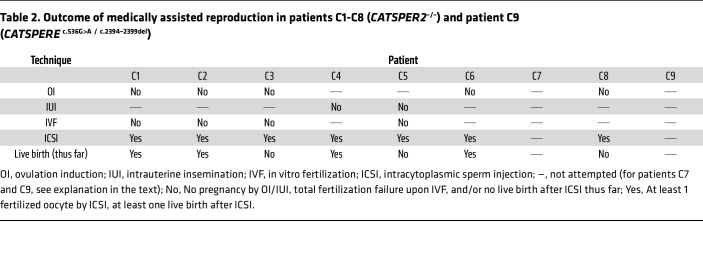
Outcome of medically assisted reproduction in patients C1-C8 (*CATSPER2*^–/–^) and patient C9 (*CATSPERE* ^c.536G>A^^/^^c.2394–2399del^)

## Version 1 01/02/2024

Electronic publication
