# Supplementary material for: Human fertilization in vivo and in vitro requires the CatSper channel to initiate sperm hyperactivation
Source: J Clin Invest. 2024 Jan 2;134(1):e173564. doi: 10.1172/JCI173564 (PMC10760960; doi:10.1172/JCI173564)
Supplement: Supplemental data [file jci-134-173564-s109.pdf]

Supplemental Material

Human fertilization in vivo and in vitro requires the CatSper channel to initiate sperm hyperactivation

Samuel Young[1]*, Christian Schiffer[1]*, Alice Wagner[1,2], Jannika Patz[1], Anton Potapenko[1], Leonie Herrmann[1], Verena Nordhoff[1], Tim Pock[1], Claudia Krallmann[1], Birgit Stallmeyer[2], Albrecht Röpke[3], Michelina Kierzek[1,4], Cristina Biagioni[1], Tao Wang[1], Lars Haalck[5], Dirk Deuster[6,#], Jan N Hansen[7], Dagmar Wachten[7], Benjamin Risse[5,8], Hermann M Behre[9], Stefan Schlatt[1], Sabine Kliesch[1], Frank Tüttelmann[2]*, Christoph Brenker[1]*, Timo Strünker[1,10]*

[1]Centre of Reproductive Medicine and Andrology, University Hospital Münster, University of Münster, Münster, Germany; [2]Institute of Reproductive Genetics, University of Münster, Münster, Germany; [3]Institute of Human Genetics, University of Münster, Münster, Germany; [4]CiM-IMPRS Graduate School, University of Münster, Münster, Germany; [5]Institute of Geoinformatics, Computer Vision and Machine Learning Systems, University of Münster, Münster, Germany; [6]Department of Phoniatrics and Pedaudiology, University Hospital Münster, University of Münster, Münster, Germany; [7]Institute of Innate Immunity, Department of Biophysical Imaging, Medical Faculty, University of Bonn, Bonn, Germany; [8]Computer Science Department, University of Münster, Münster, Germany; [9]UKM Fertility Centre, University Hospital Münster, Münster, Germany; [10]Cells in Motion Interfaculty Centre, University of Münster, Münster. [#]deceased
*these authors contributed equally to the work

Corresponding authors:

Timo Strünker
Centre of Reproductive Medicine and Andrology
Albert-Schweitzer-Campus 1, Geb. D11
48149 Münster
Phone: +49-251-83-58238
Email: timo.struenker@ukmuenster.de

Frank Tüttelmann
Institute of Reproductive Genetics
Vesaliusweg 12-14,
48149 Münster
Phone: + 49-251-83-55411
Email: frank.tuettelmann@ukmuenster.de

Supplemental Figures

Supplementary Figure 1. Single-cell imaging of progesterone-evoked Ca^{2+} signals in control and *CATSPER2*$^{-/-}$ sperm.

Supplementary Figure 2. Iterative improvements of the CatSper-Activity-Test.

Supplementary Figure 3. MLPA data at the chromosome 15q15.3 locus of the eight *CATSPER2*$^{-/-}$ patients.

Supplementary Figure 4. Stratification of men enrolled in the study and estimation of prevalences for CatSper-related male-factor infertility.

Supplementary Figure 5. Audiometric analysis of six *CATSPER2*$^{-/-}$ patients

Supplementary Figure 6. Spontaneous hyperactivation of control and *CATSPER2*$^{-/-}$ sperm.

Supplementary Figure 7. Kinetic CASA.

Supplementary Figure 8. Frequency and amplitude of the flagellar beat and the rotation velocity of control sperm from donors and *CATSPER2*$^{-/-}$ sperm from patient C1 (see Figure 7, A and B).

Supplementary Figure 9. Patch-clamp recordings and basal-motility analysis of sperm from a *CATSPER2*$^{+/-}$ patient.

Supplemental Tables

Supplementary Table 1. CatSper channel subunits with the corresponding murine and human gene names.

Supplementary Table 2. Results of SNP-array analysis.

Supplementary Table 3: History of medically assisted reproduction of the *CATSPER2*$^{-/-}$ patients C1-C8 and the *CATSPERE*$^{mut/del}$ patient C9

Supplementary Table 4. Primer sequences used for Sanger validation of *CATSPERE* variants in patient C9.

Supplementary Movies (see separate movie files)

Supplementary Movie 1. Representative video of the ejaculate of a *CATSPER2*$^{-/-}$ patient

Supplementary Movie 2. Video of an IVF attempt with sperm from the *CATSPER2*$^{-/-}$ patient C5

Supplementary Movie 3. Video of head-tethered control sperm before and after uncaging progesterone

Supplementary Movie 4. Video of head-tethered *CATSPER2*$^{-/-}$ sperm before and after uncaging progesterone

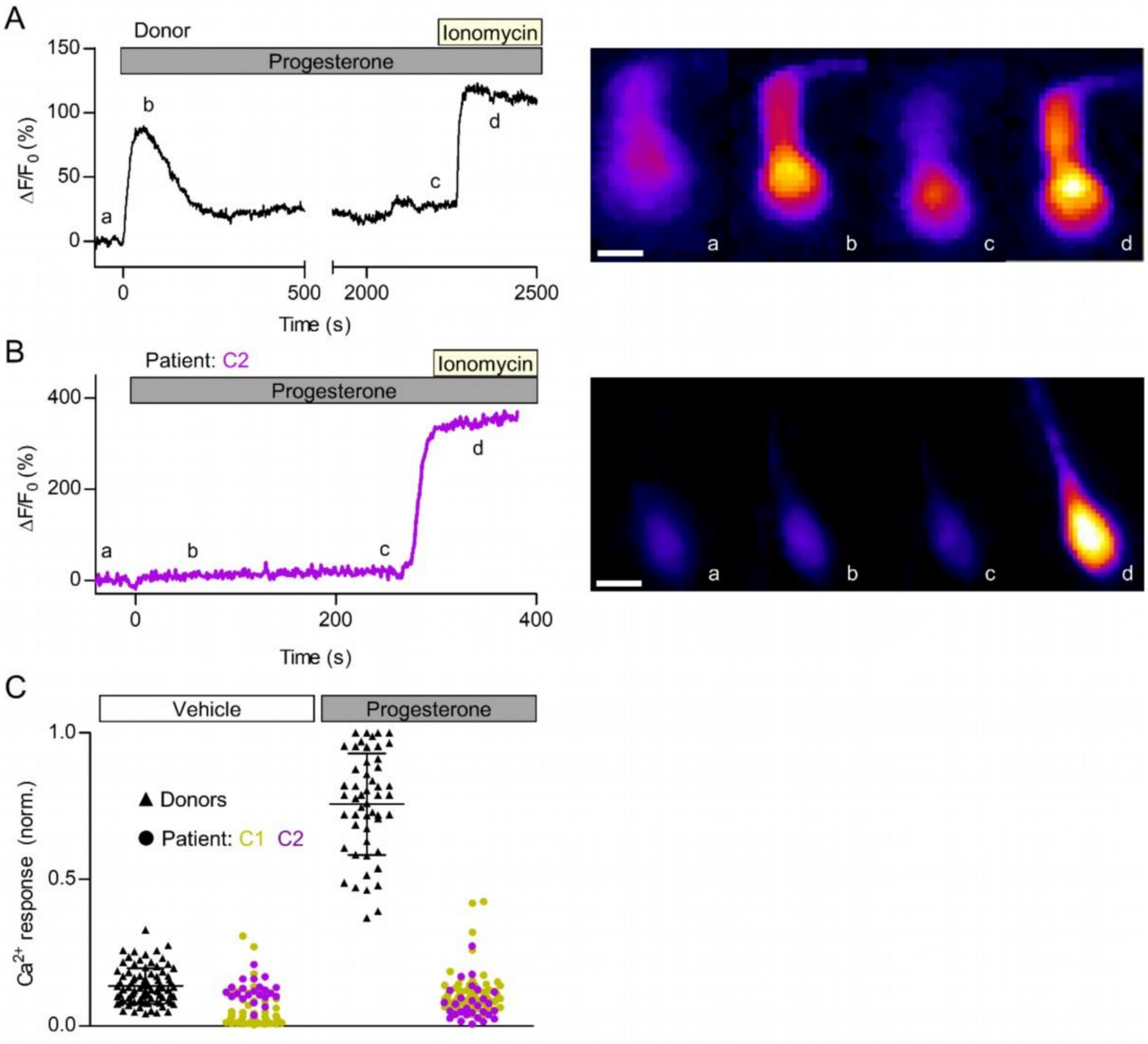

Supplementary Figure 1. Single-cell imaging of progesterone-evoked Ca^{2+} signals in control and *CATSPER2$^{-/-}$* sperm. (A) Left: Representative Ca^{2+} response evoked by progesterone (applied at t = 0) and, subsequently, ionomycin in an immobilized sperm cell from a donor loaded with the Ca^{2+} indicator Fluo-4. The signal is displayed as $\Delta F/F_0$ (%), i.e. the change in fluorescence relative to the mean basal fluorescence (F_0) of the ≥ 60 images before application of progesterone. Right: False-color coded fluorescence images taken at the indicated time points (a-d), i.e. before and at the peak of the progesterone- and ionomycin-induced Ca^{2+} signal, respectively. **(B)** Left: Representative Ca^{2+} response evoked by progesterone (applied at t = 0) and, subsequently, ionomycin in an immobilized *CATSPER2$^{-/-}$* sperm cell from patient C2. Right: False-color coded fluorescence images taken at the indicated time points (a-d). **(C)** Scatter plot (mean ± SD) of the maximal signal amplitude evoked by application of buffer or progesterone in control (black, buffer and progesterone n = 86 and n = 51 sperm, respectively) and *CATSPER2$^{-/-}$* sperm (color-coded, buffer and progesterone n = 84 and n = 91 sperm, respectively) from patients C1 and C2, relative to that evoked by ionomycin (set to 1). Scale bars represent 10 µm.

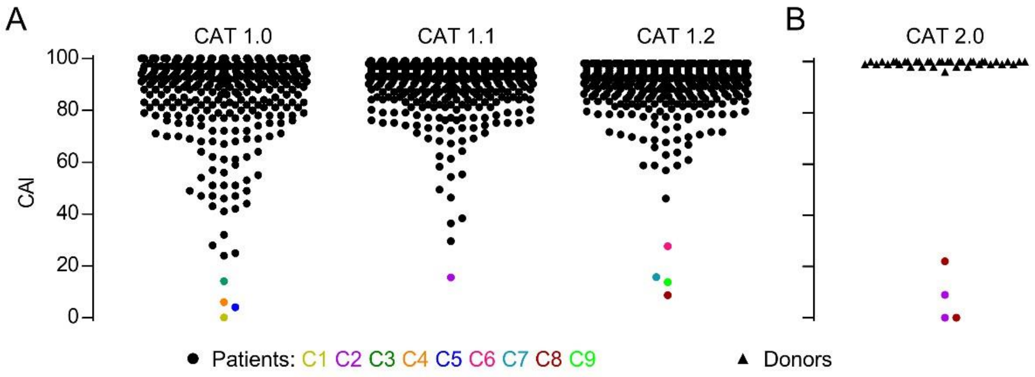

Supplementary Figure 2. Iterative improvements of the CatSper-Activity-Test. (A) Aggregated CatSper-Activity-Test data shown in Figure 1C separated by distinct refinement steps: CAI values from semen samples of men undergoing semen analysis determined 15 minutes (CAT 1.0, n = 370) or 30 minutes (CAT 1.1, n = 540) after dilution in Buffer A and Buffer B, or 30 minutes after dilution in Buffer B also containing EDTA (CAT 1.2, n = 1376). Patients with confirmed impaired or loss of CatSper function are labeled C1-C9 and indicated with a colored-coded circle. **(B)** CAI values determined 60 minutes after dilution of semen samples from donors (black triangles, n = 38) and patients C2 and C8 (color-coded circles, n = 4) in Buffer A and Buffer B containing EDTA.

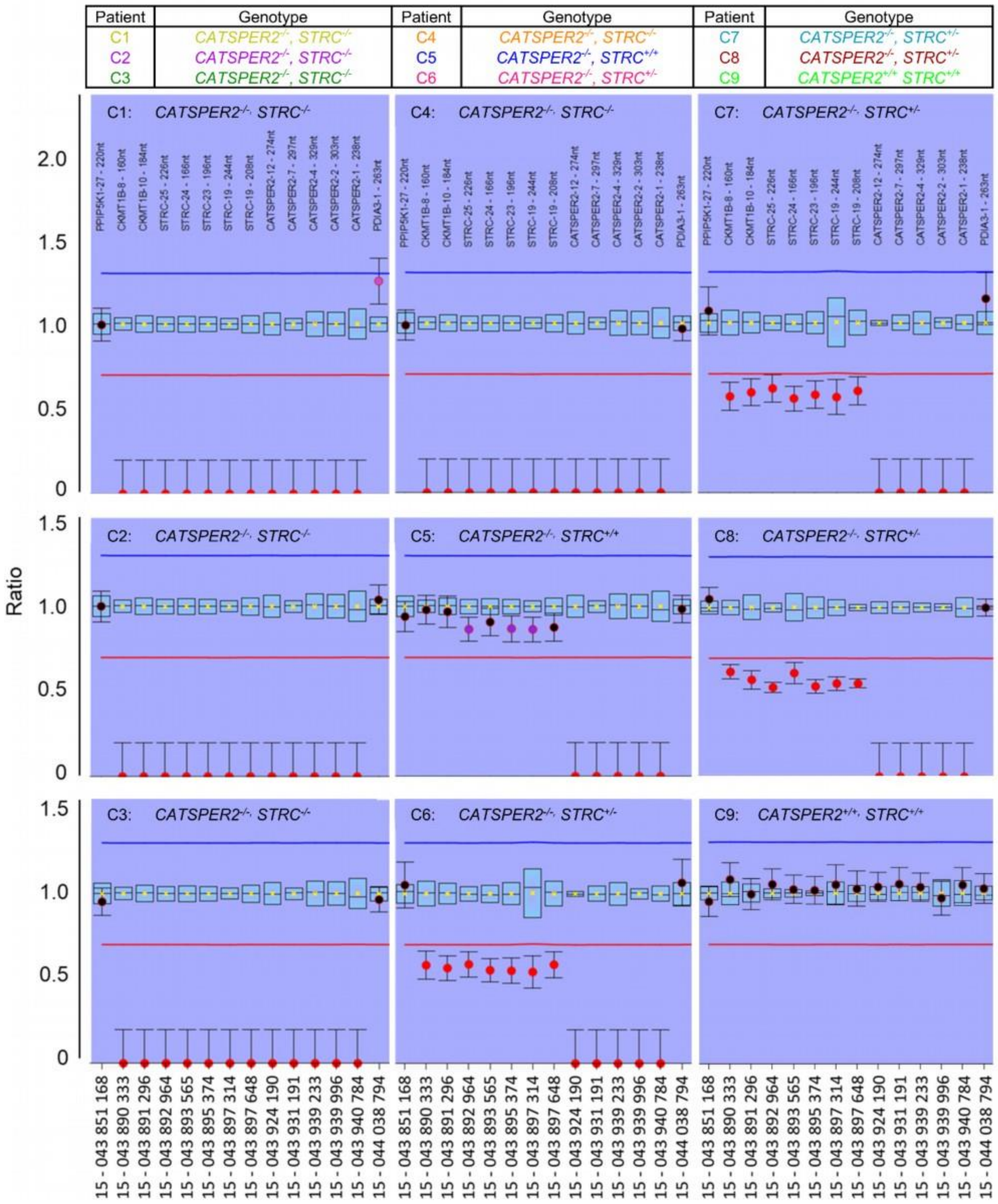

Supplementary Figure 3. MLPA data at the chromosome 15q15.3 locus of the eight *CATSPER2*$^{-/-}$ patients. Two flanking probes encompass reference probes for *CATSPER2*, *STRC*, and *CKMT1B*. The ratio (y-axis, range: 2.0–0) is indicative of the copy number status (normal = 0.8–1.2, heterozygous deletion = 0.4–0.7, homozygous deletion = 0). Red circles (mean ± SD) represent the patient DNA samples. Positive control DNA samples are represented as high-low box plots.

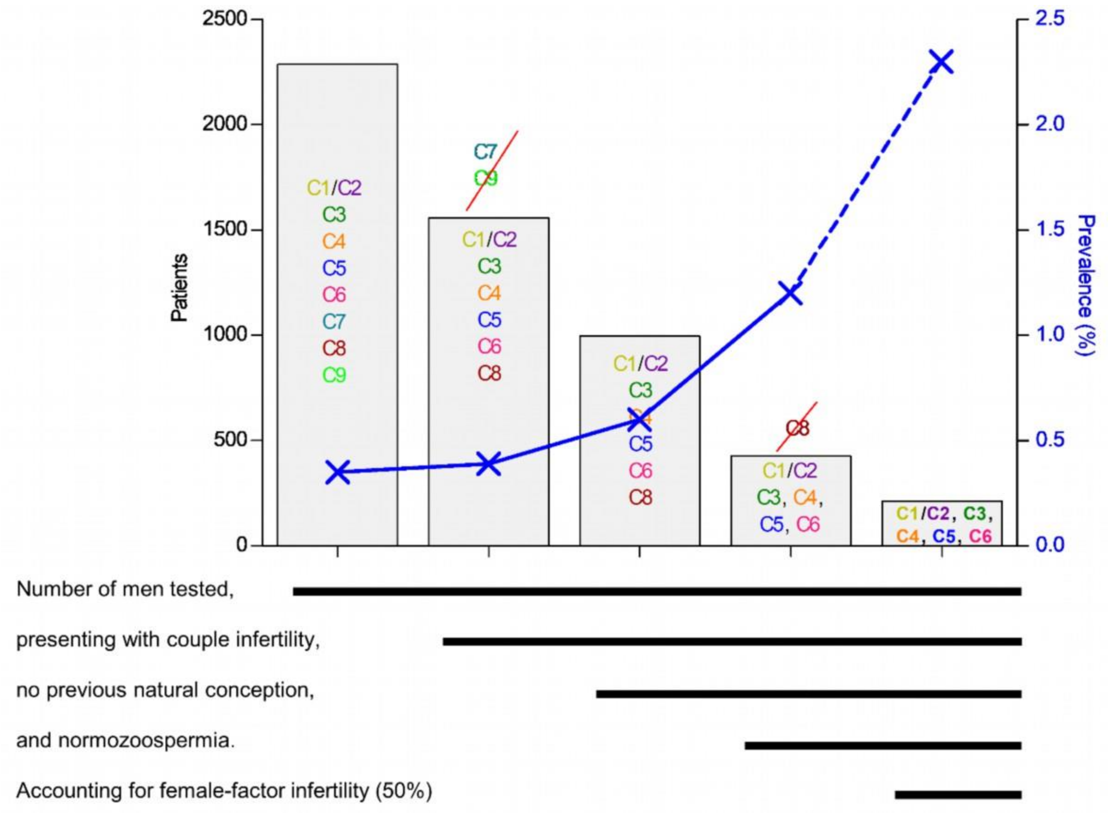

Supplementary Figure 4. Stratification of men enrolled in the study and estimation of prevalences for CatSper-related male-factor infertility. Among a total of 2,286 patients enrolled, eight patients with loss of (C1-C8) and one (C9) with severely impaired CatSper function were identified. Among them were two brothers (C1 and C2) counting as one for the calculation of prevalence (blue, right y-axis). Of these, 1,557 patients, but not patient C7 and C9, presented because of couple infertility, 997 of which with no previous natural conception, of which 426 were also normozoospermic (excluding patient C8). Assuming that for about 50% of these men/couples the infertility is rather due to a female factor, we can estimate a prevalence of 2.3% for a CatSper-related male-factor infertility (gray box / dashed line) among couples presenting with unexplained infertility.

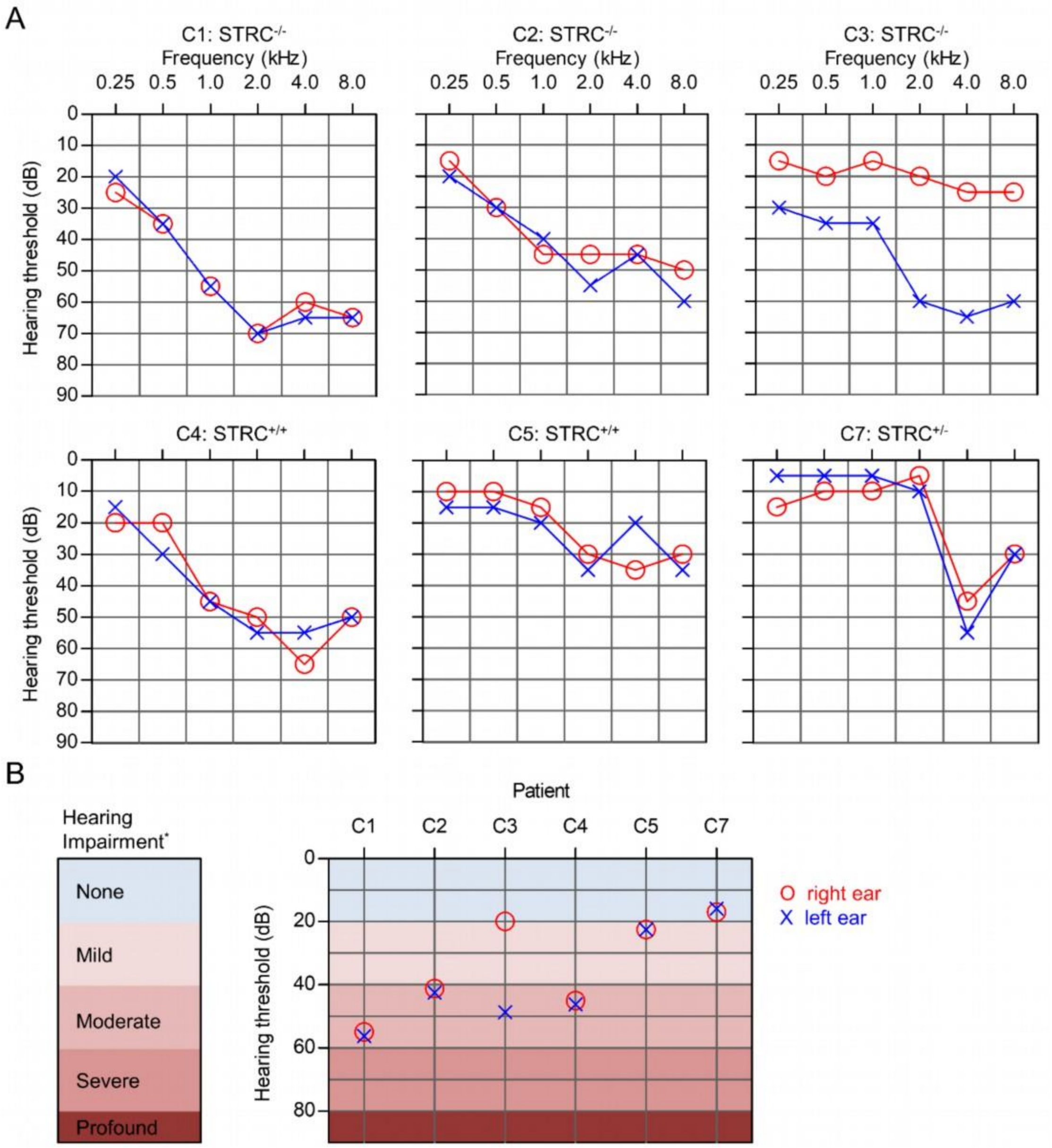

Supplementary Figure 5. Audiometric analysis of six *CATSPER2*$^{-/-}$ patients: (A) Audiograms depicting the hearing threshold of a sound at a given frequency in the right (red circles) and left ear (blue crosses). *STRC*$^{-/-}$ patients (i.e. patients C1, C2, C3, and C4) characteristically had sloping, high-frequency hearing impairment. **(B)** Summary of the audiometric analysis and scoring of the hearing impairments. Loss of *STRC* translated into moderate hearing loss, except in the case of patient C3, who suffered from moderate, unilateral hearing loss. Patient C7 that was heterozygous for *STRC* (*STRC*$^{+/-}$) and patient C5 with unaffected *STRC* presented with normal hearing. Hearing impairment is defined by the WHO as the averaged hearing threshold at 500, 1000, 2000, and 4000 Hz.

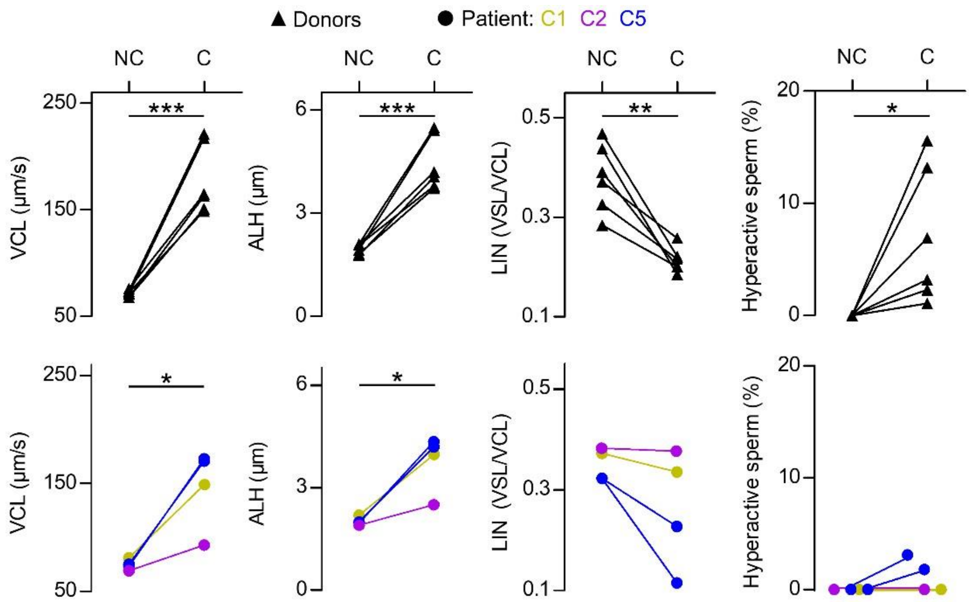

Supplementary Figure 6. Spontaneous hyperactivation of control and *CATSPER2*$^{-/-}$ sperm. Paired plots of kinematic parameters and fraction of hyperactivated sperm from donors (black triangles, n = 6) and *CATSPER2*$^{-/-}$ patients (color-coded circles, n = 4, i.e., four independent experiments with sperm from patients C1, C2, C5) incubated in parallel for at least three hours under non-capacitating (NC) and capacitating (C) conditions, respectively. These paired comparisons indicate that both in donors and *CATSPER2*$^{-/-}$ patients, capacitation affected CASA parameters when averaged over the entire sperm population. However, on the single sperm level, only in donors but not in *CATSPER2*$^{-/-}$ patients, a significant fraction of sperm exceeds the hyperactive-motility threshold upon capacitation. *P < 0.05, **P < 0.01***P < 0.001, paired t-test.

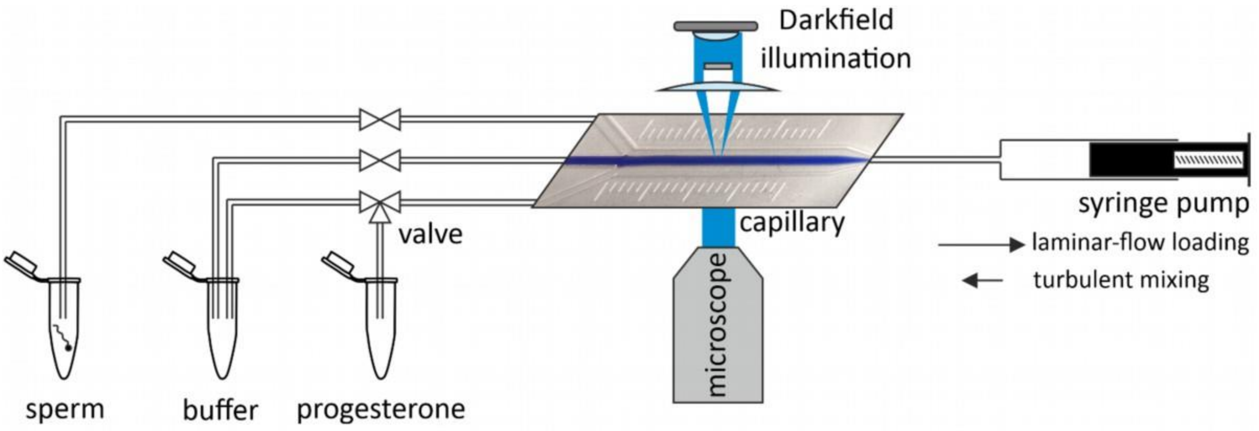

Supplementary Figure 7. Kinetic CASA. Illustration of the microfluidic set-up allowing for rapid mixing of sperm with progesterone and time-resolved analysis of ensuing motility responses by CASA. In brief, parallel laminar flows of two different solutions were established in a microcapillary. Using a syringe pump, sperm in HTF^{++} were pulled through the capillary along with HTF^{++} containing progesterone or HTF^{++} alone. Upon brief reversal of the flow direction, the solutions were rapidly mixed and the flow was stopped. Subsequently, every 15 seconds, the fraction of hyperactive sperm was determined by CASA and corrected for the basal level determined upon mixing of sperm with HTF^{++} alone.

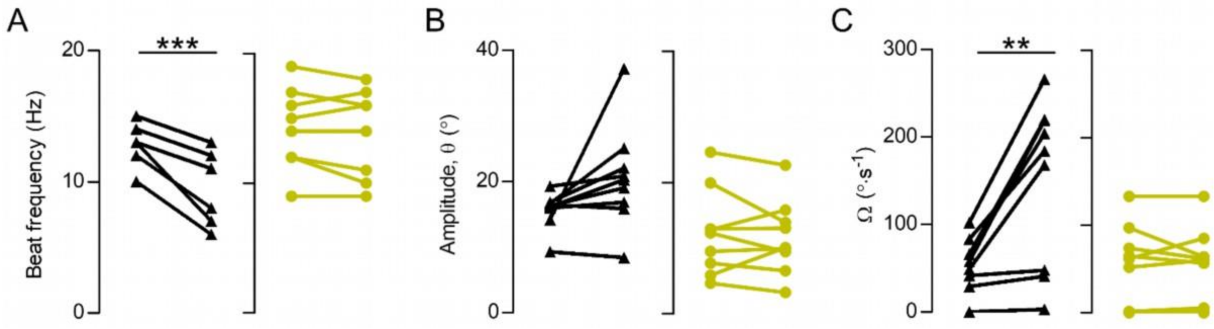

Supplementary Figure 8. Frequency and amplitude of the flagellar beat and the rotation velocity of control sperm from donors and *CATSPER2*$^{-/-}$ sperm from patient C1 (see Figure 7). (A-C) Paired plots with mean ± SD depicting the beat frequency (A), beat amplitude, θ (B), and rotation velocity Ω(°·s^{-1}) (C) of sperm from donors (black, control, n = 9) and patient C1 (gold, n = 9) before and after uncaging progesterone. **P < 0.01, ***P < 0.001, paired t-test.

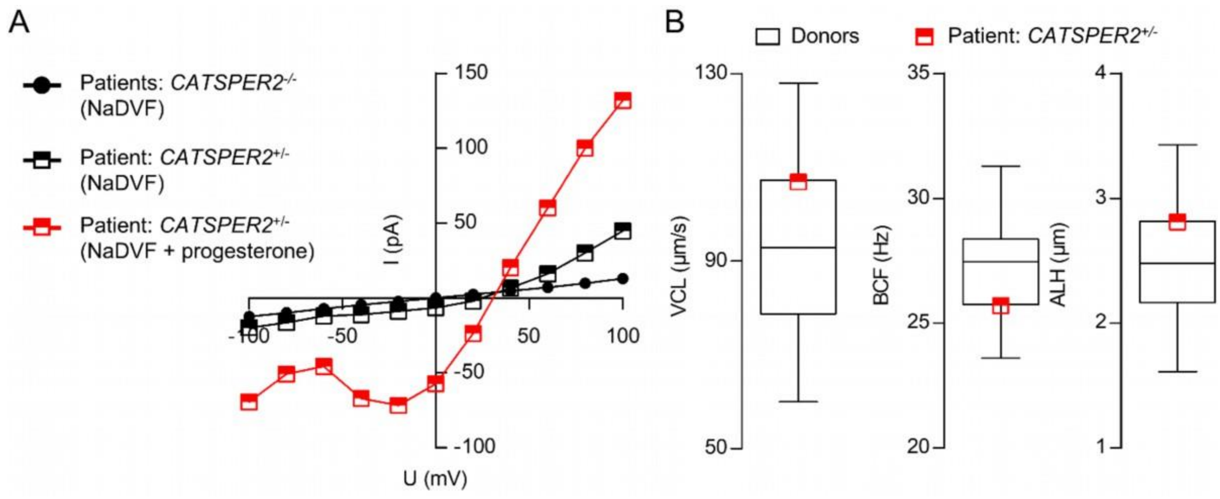

Supplementary Figure 9. Patch-clamp recordings and basal-motility analysis of sperm from a *CATSPER2*$^{+/-}$ proven father. (A) Current-voltage relationship of monovalent currents in a sperm cell from a *CATSPER2*$^{+/-}$ men perfused with divalent-free Na^{+}-based solution (NaDVF) (half-filled black squares) and NaDVF fortified with progesterone (2 µM) (half-filled red squares), and monovalent currents in sperm cells from *CATSPER2*$^{-/-}$ patients perfused with NaDVF (black circles) (mean ± SD, n = 5). The membrane voltage was stepped from -100 to +100 in increments of 10 mV from a holding potential of -80 mV. **(B)** Box and whiskers plots (whiskers: min to max) of kinematic parameters of control sperm from donors depicted in Figure 5 (black, n = 22) and from a *CATSPER*$^{+/-}$ patient (half-filled red squares).

Supplementary Table 1. CatSper channel subunits with the corresponding murine and human gene names.

**CatSper subunit (protein)**	**Gene name (murine)**	**Gene name (human)**
CatSper1	*Catsper1*	*CATSPER1*
CatSper2	*Catsper2*	*CATSPER2*
CatSper3	*Catsper3*	*CATSPER3*
CatSper4	*Catsper4*	*CATSPER4*
CatSperβ	*Catsperb*	*CATSPERB*
CatSperγ	*Catsperg*	*CATSPERG*
CatSperδ	*Catsperd*	*CATSPERD*
CatSperε	*Catspere*	*CATSPERE*
CatSperζ	*Catsperz*	*CATSPERZ*
CatSperη	*Catsperh*	*CATSPERH*
CatSperт	*Catspert*	*CATSPERT*
Efcab9	*Efcab9*	*EFCAB9*
Slco6c1	*Slco6c1*	*SLCO6A1* (ortholog)
Tmem249	*Tmem249*	*TMEM249*
Trim69	*Trim69*	*TRIM69*

Supplementary Table 2. Results of SNP-array analysis.

Patient	Allele	Chromosome	Start	End	Size (bp)	*CATSPER2* deletion/zygosity
C1	Allele 1	15q15.3	43,892,807	43,939,642	46,836	homozygous
	Allele 2	15q15.3	43,892,807	43,939,642	46,836	
C2	Allele 1	15q15.3	43,892,807	43,939,642	46,836	homozygous
	Allele 2	15q15.3	43,892,807	43,939,642	46,836	
C3	Allele 1	15q15.3	43,892,807	43,939,642	46,836	homozygous
	Allele 2	15q15.3	43,892,807	43,939,642	46,836	
C4	Allele 1	15q15.3	43,892,807	43,939,642	46,836	homozygous
	Allele 2	15q15.3	43,892,807	43,939,642	46,836	
C5	Allele 1	15q15.3	43,916,071	43,939,642	23,572	homozygous
	Allele 2	15q15.3	43,916,071	43,939,642	23,572	
C6	Allele 1	15q15.3	43,916,071	43,939,642	23,572	compound heterozygous
	Allele 2	15q15.3	43,892,807	43,939,642	46,836	
C7	Allele 1	15q15.3	43,916,071	43,939,642	23,572	compound heterozygous
	Allele 2	15q15.3	43,892,807	43,939,642	46,836	
C8	Allele 1	15q15.3	43,916,071	43,939,642	23,572	compound heterozygous
	Allele 2	15q15.3	43,892,807	43,939,642	46,836	
C9	no deletion					

Supplementary Table 3: History of medically assisted reproduction of the *CATSPER2*$^{-/-}$ patients C1-C8 and the *CATSPERE*$^{mut/del}$ patient C9

Patient	Length of infertility (years)[A]	OI	IUI	IVF	ICSI
C1	2	3 attempts: no pregnancy	-	1 attempt: 10 oocytes, total fertilization failure	3 attempts: 16 oocytes, 9 fertilized, 2 pregnancies with live births of healthy children
C2	2	>15 attempts: no pregnancy	-	1 attempt: 4 oocytes, total fertilization failure	2 attempts: 34 oocytes, 26 fertilized, 2 pregnancies with live birth of healthy children
C3	8	1 attempt: no pregnancy	-	1 attempt: 3 oocytes, total fertilization failure	3 attempts: 5 oocytes, 3 fertilized no pregnancy
C4	2	-	1 attempt: no pregnancy	-	1 attempt: details not available, pregancy with live birth of healthy twins
C5	1	-	2 attempts: no pregnancy	1 attempt: 5 oocytes, total fertilization failure	4 attempts: 40 oocytes, 25 fertilized, no pregnancy
C6	1	2 attempts: no pregnancy	-	-	5 attempts: 4 oocytes, 1 fertilized, 1 pregnancy with live birth of healthy child
C7	See explanation in the text	-	-	-	-
C8	1	2 attempts: no pregnancy	-	-	3 attempts: 24 oocytes, 16 fertilized no pregnancy
C9	See explanation in the text	-	-	-	-

[A]Period of unprotected regular intercourse without pregnancy before first presenting at a fertility center.

Supplementary Table 4. Primer sequences used for Sanger validation of *CATSPERE* variants in patient C9.

**Primer**	**Sequence 5´-3´**
CATSPERE EX8_F	GCT GAA TCT TGA GCA CAT GGT AAA TTT
CATSPERE EX8_R	AGG ATT TGC CAC CAA CCT GA
CATSPERE EX18F	GCT GGA ACT CTT AAC CCC TCT
CATSPERE EX18R	TCC TAC CCA CTG CTG CCT TA
